# An Integrated Modelling Framework to Determine Terrestrial Carbon Dioxide Removal via Enhanced Rock Weathering

**DOI:** 10.1111/gcb.70650

**Published:** 2025-12-17

**Authors:** Ziyan Zhang, Gregory Jones, Salvatore Calabrese, Matteo Bertagni, Simone Fatichi, Bonnie Waring, Athanasios Paschalis

**Affiliations:** ^1^ Department of Civil and Environmental Engineering Imperial College London London UK; ^2^ Department of Life Sciences Imperial College London Ascot UK; ^3^ Department of Biological and Agricultural Engineering Texas A&M University College Station Texas USA; ^4^ Department of Environment, Land and Infrastructure Engineering Politecnico di Torino Turin Italy; ^5^ Department of Civil and Environmental Engineering National University of Singapore Singapore Singapore; ^6^ Department of Civil and Environmental Engineering University of Cyprus Nicosia Cyprus

**Keywords:** biogeochemical modelling, carbon dioxide removal, climate change mitigation, ecohydrology, enhanced rock weathering, plant–soil interactions, reactive transport

## Abstract

Enhanced rock weathering (ERW) is an emerging carbon dioxide removal (CDR) strategy that can support net‐zero emission targets. However, current ERW modelling efforts rely on assumptions that introduce substantial variation in CDR estimates across varying ecosystems and hydroclimatic conditions. They typically ignore or oversimplify plant–soil interactions and high‐frequency hydrological dynamics, obscuring short‐term weathering responses and biotic feedbacks to soil moisture dynamics. Here, we introduce an integrated, process‐based modelling framework, T&C‐SMEW, which represents ecohydrological and ERW dynamics, along with microbially explicit biogeochemical processes. We compared framework simulations against a controlled mesocosm experiment and long‐term field observations, demonstrating its ability to reproduce feedstock cation release, soil pH dynamics, gross primary production, and CO_2_ fluxes. T&C‐SMEW reveals hydrological constraints and vegetation effects on ERW‐mediated CDR by quantifying impacts on ecosystem respiration, net ecosystem exchange, and alkalinity export, emphasising the importance of ecohydrological modelling for ecosystem‐level CDR estimation. These advances provide a modelling framework for identifying optimal deployment scenarios to establish ERW as a viable and operationally feasible CDR approach.

## Graphical abstract

T&C‐SMEW is an integrated modelling framework that couples ecohydrological, biogeochemical, and weathering processes to quantify carbon dioxide removal (CDR) via enhanced rock weathering (ERW). The model captures high‐frequency plant–soil–water interactions that govern cation release, alkalinity generation, soil pH dynamics, and ecosystem carbon fluxes. Comparisons with mesocosm and field data demonstrate that hydrological variability, vegetation activity, and feedstock characteristics strongly influence the effectiveness of ERW as a CDR strategy, providing a basis for assessing deployment potential under real‐world conditions.

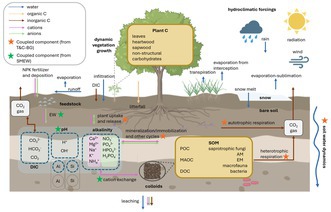

## 1 Introduction

Human activities increase atmospheric greenhouse gas levels, intensifying climate change and resulting in widespread risks to society and the environment (Hansen et al. 2013). Reducing these risks requires the urgent and widespread adoption of approaches to reduce emissions, alongside CO_2_ removal (CDR) strategies to offset residual emissions (Gasser et al. 2015; Riahi et al. 2022). International CDR targets (Calvin et al. 2023; United Nations Environment Programme 2023), involving the rapid implementation of CDR strategies, suggest target sequestration of 7–9 Gt CO_2_ year^−1^ by 2050 (equivalent to 19%–14% of global CO_2_ emissions in 2023) (Hansen et al. 2017; International Energy Agency 2024; S. Smith et al. 2023).

There are several options for large‐scale CDR, for example, re/afforestation, bioenergy with carbon capture and storage (BECCS), and direct air capture (DAC) (Smith et al. 2015). BECCS and re/afforestation require large land areas (Abdalqadir et al. 2024), which may have strong negative impacts on biodiversity and food security (Deprez et al. 2024); while DAC is costly, energy‐intensive, and therefore not yet widely adopted (Young et al. 2023). Another promising option is enhanced rock weathering (ERW), which has gained attention as a potentially scalable terrestrial CDR strategy that also improves other ecosystem components such as soil fertility (Skov et al. 2024), biomass production (Battles et al. 2014; Taylor et al. 2021), and crop yields (Beerling et al. 2024; Kantola et al. 2023). ERW is a CDR technique that accelerates natural silicate weathering by amending soils with finely ground Ca‐ and Mg‐rich silicate feedstock, such as basalt, olivine, and wollastonite (Hartmann et al. 2013). Rock weathering releases base cations (Mg^2+^, Ca^2+^, K^+^, Na^+^) that promote the conversion of atmospheric CO_2_ into dissolved inorganic C (DIC: primarily in the form of HCO_3_^−^). Depending on soil moisture and drainage dynamics, weathering products may form pedogenic carbonates in the soil or be transported to deep aquifers, rivers, and ultimately, the ocean, where sequestered C is stable for millennia (Renforth and Henderson 2017). ERW has the potential to globally sequester up to 4.4 Gt CO_2_ year^−1^ (Gaucher et al. 2025), acting as an important option in the portfolio of CDR strategies necessary to achieve international climate targets. Importantly, ERW is scalable using existing agricultural infrastructure and can utilise relatively low‐cost and abundant silicate rock sources or industrial waste, such as steel slag (Beerling et al. 2020; Renforth et al. 2011).

Numerical modelling is key to understanding complex ERW dynamics and estimating its CDR potential across varying spatial and temporal scales (Beerling et al. 2020, 2025; Bertagni et al. 2025; Kantzas et al. 2022; Kanzaki et al. 2022, 2024). The efficacy of ERW for CDR is linked to plant, soil, and hydrological processes, which govern the interactions between feedstock weathering, plant growth, and soil biogeochemical dynamics (Beerling et al. 2018; Calabrese and Porporato 2020). However, representations of plant and hydrological dynamics often rely on simplified assumptions by ignoring plant activity (Kanzaki et al. 2024) and high‐frequency hydrological dynamics (Kantzas et al. 2022). These simplifications mask the short temporal scale, non‐linear responses of weathering, soil C, and biological processes, which respond to changes in soil moisture and can lead to substantial variations in CDR potential under different hydroclimatic regimes (Goddéris et al. 2006; Porporato et al. 2003). For example, autotrophic and heterotrophic respiration serve as an additional source of CO_2_, contributing to the production of carbonic acid. Base cations also act as nutrients for plant growth, potentially enhancing primary production, and functioning as a dominant pathway for ecosystem‐scale CDR (e.g., Battles et al. 2014; Taylor et al. 2021). Plant uptake of cations depletes pore water concentrations by acting as an alkalinity sink, producing acidity in the soil solution and driving weathering reactions. Variable effects of ERW on short‐term soil organic C (SOC) mineralisation have also been observed, with some studies reporting increased soil CO_2_ fluxes (Yan et al. 2023), while others indicate reductions or no changes (Dietzen et al. 2018). Therefore, models using such simplifications may struggle to represent the true variability and driving factors of ERW processes across diverse ecosystems and hydroclimates (Cipolla et al. 2021a; Roelandt et al. 2010). Due to the interactions between local hydrological processes, plant dynamics, nutrient cycling, and feedstock weathering rates, incorporating these complexities into state‐of‐the‐art models is key to accurately capturing the CDR potential of ERW in real‐world systems (Amann et al. 2020; Buckingham and Henderson 2023).

Diverse ERW modelling strategies incorporate feedstock properties, such as mineralogy, chemistry, and physical characteristics, as key input parameters in reactive transport models to trace the fate of solid, aqueous, and gaseous phases related to feedstock dissolution and the transport of weathering products (e.g., Kantzas et al. 2022; Kanzaki et al. 2024; Bertagni et al. 2025). Here, we introduce a novel integrated modelling framework, T&C‐SMEW, which mechanistically couples the microbially explicit ecohydrological model, T&C‐BG (Fatichi et al. 2012, 2019), with the depth‐averaged reactive transport model, SMEW (Bertagni et al. 2025), which resolves ecohydrological and biogeochemical dynamics, including ERW processes. While SMEW has demonstrated the ability to capture ERW dynamics, T&C‐BG further provides a detailed representation of coupled vegetation, nutrient cycling and hydrological dynamics (Fatichi et al. 2012, 2019, 2016; Fatichi and Ivanov 2014; Pappas et al. 2016; Mastrotheodoros et al. 2017; Paschalis et al. 2024). By integrating these two models, the T&C‐SMEW framework captures the critical interplay between vegetation growth, hydrological dynamics, pore water chemistry, and mafic feedstock dissolution related to ERW at the field scale.

T&C‐SMEW adopts a coupled structure, combining a one‐dimensional ecohydrology component with vertically lumped soil biogeochemical and ERW modules. This design reduces computational demand by avoiding the need to explicitly resolve vertical concentration gradients, while still representing depth‐integrated soil biogeochemical processes and retaining hydrological variability that drives them. This integrated framework aims to capture the effectiveness and variability of ERW across temporal scales, hydroclimatic regimes, and management contexts, thereby supporting an improved understanding of ERW as a scalable CDR strategy.

Overall, this study aims to evaluate model performance across increasing levels of system complexity, with the aim of representing ecosystem‐scale hydrological, biogeochemical, and weathering dynamics, thereby revealing its operational strengths and limitations in predicting ecosystem responses to ERW.

## 2 Model Description

**FIGURE 1:**
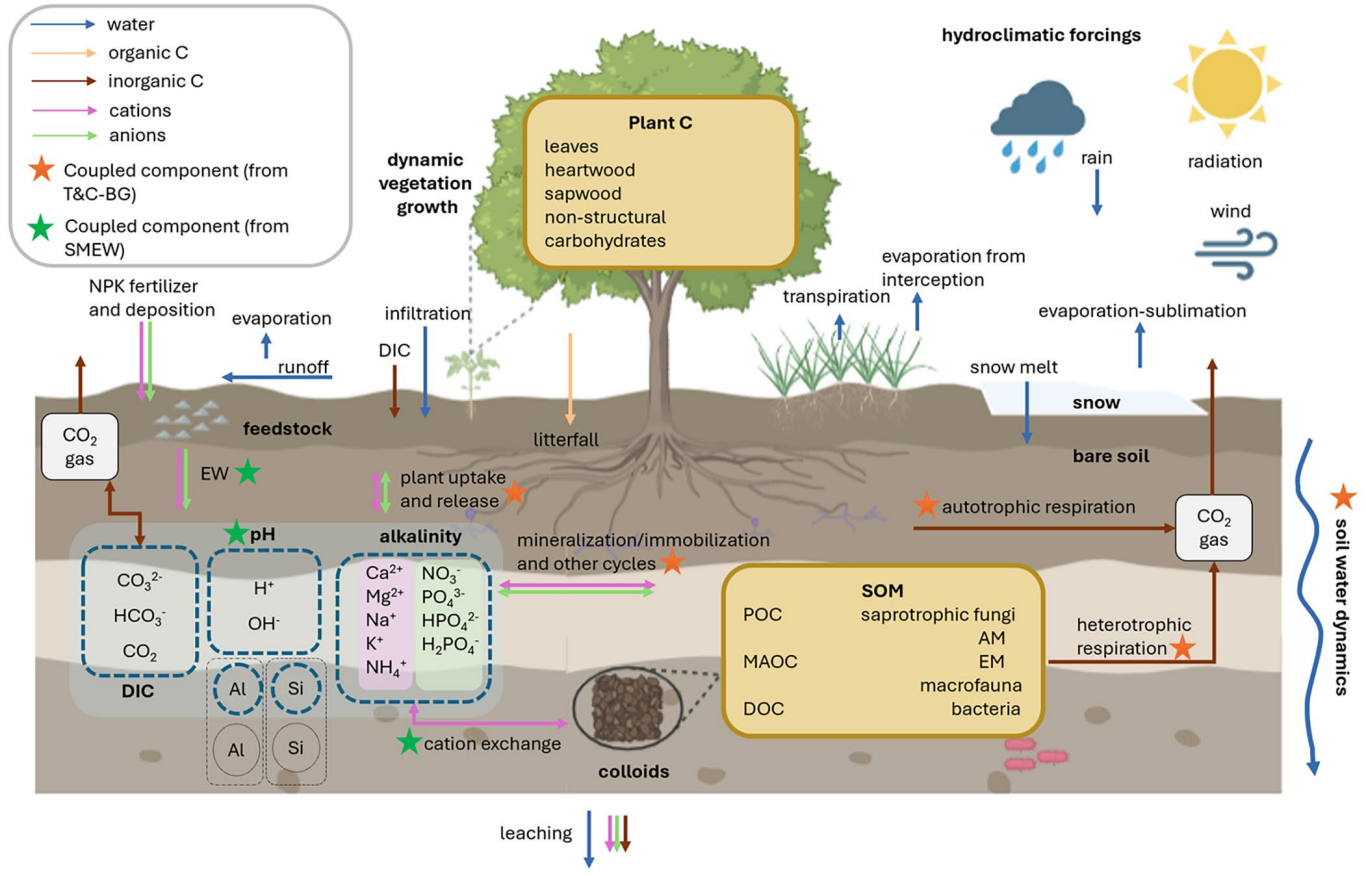
The T&C‐SMEW model framework. The coloured arrows represent material flows associated with enhanced rock weathering (ERW): Water (blue), organic C (peach), inorganic C (brown), cations (pink), and anions (green). Orange stars denote components originating from the ecohydrological model, Tethys & Chloris‐Biogeochemistry (T&C‐BG; Fatichi et al. 2019), coupled to the reactive transport model, including ERW‐related pathways (SMEW; Bertagni et al. 2025). Green stars represent components originating from SMEW and integrated with T&C‐BG. The following abbreviations are used in the diagram: AM, arbuscular mycorrhiza; EM, ectomycorrhiza; DIC, dissolved inorganic carbon; DOC, dissolved organic carbon; MAOC, mineral‐associated organic carbon; POC, particulate organic carbon.

T&C‐SMEW resolves the C, water, and energy budgets alongside nutrient and ERW dynamics at the plot/field scale (Figure 1). By combining mechanistic representations of vegetation‐soil‐hydrology interactions with depth‐integrated ERW processes, the model enables the assessment of ERW across varying ecosystems and hydroclimatic conditions. This includes quantifying CO_2_ sequestration potential through alkalinity generation, assessing feedbacks on plant productivity, and evaluating potential environmental trade‐offs, such as excessive nutrient leaching. Key integrated dynamics and equations are presented in Sections 2.1, 2.4, while remaining dynamics that follow the original models can be found in Fatichi et al. (2019) and Bertagni et al. (2025).

Hourly mass and energy fluxes (Section 2.1) determine daily dynamics of vegetation and soil C, which influence land‐atmosphere feedback processes through alterations to plant biophysics and physiology, as represented in T&C‐BG (Fatichi et al. 2019). Plant structural changes (Section 2.2), such as the dynamic evolution of leaf area index and physiological responses, including stomatal conductance, respond to environmental cues that can modify water, C, and nutrient exchange, as well as feedback on vegetation growth. Soil C and nutrient fluxes (Section 2.3) also respond to changes in environmental conditions, such as plant nutrient demand, soil pH and cation exchange processes.

T&C‐SMEW simulates ERW processes (adapted from SMEW and hereafter referred to as the ERW module; Section 2.4), including alkalinity generation, pH dynamics and DIC cycling, and represents the cycling of common elements present within mafic feedstock, which are essential to plant growth and involved in major soil biogeochemical processes (Mg, Ca, K, Na, P, Si). Additionally, the explicit representation of anthropogenic activities that influence NO_3_^−^, NH_4_^+^, and mineral phosphorus species (PO_4_^3−^, HPO_4_^2−^, H_2_PO_4_^−^), such as fertiliser application, enables insights into strong acid weathering and perturbations to CDR‐related weathering fluxes.

T&C‐SMEW also considers ecosystem‐specific management regimes, incorporating forest management practices such as logging and fire control (Fatichi et al. 2019). Grazing and fertilisation also apply to agricultural settings (Buckley Paules et al. 2025).

### 2.1 Water and Energy Dynamics

T&C‐SMEW computes the hourly energy balance as the exchange of radiation, heat, and moisture between the land surface and atmosphere (Fatichi et al. 2012). Net radiation is calculated as the sum of absorbed shortwave and longwave radiation, which is combined with advective heat from precipitation and partitioned into sensible, latent, and ground heat fluxes to ensure energy balance closure at each time step. Factors influencing energy partition include hydrological dynamics, such as those that affect soil moisture, which can lead to plant water stress. This leads to stomatal closure on short time scales and changes in vegetation structure at longer time scales. In turn, alterations to vegetation structure influence surface temperature and subsurface heat transfer.

Hydrological dynamics are resolved at an hourly timescale by modelling direct rainfall onto non‐vegetated areas, throughfall among two vegetation layers, snowmelt, dew, intercepted water drainage, external runoff, and evapotranspiration. Based on the magnitude and intensity of water influx and preceding soil moisture conditions, the water flux will infiltrate or be either partially or entirely excluded as surface runoff (Brutsaert 2005; Panday and Huyakorn 2004). Soil moisture dynamics, crucial for resolving fine‐resolution temporal weathering processes, are determined by solving the 1D Richards equation using a finite volume discretisation (Fatichi et al. 2012).

### 2.2 Vegetation Dynamics

Vegetation structure is conceptualised as two layers, high and low stature, where high vegetation can shade the lower based on the two‐stream approximation (Dai et al. 2004; Dickinson 1983; Fatichi et al. 2012). Plant traits are defined by lifeform, physiology, and structural attributes (Fatichi et al. 2012). Mass and energy fluxes drive the daily C dynamics of vegetation by controlling photosynthetic activity and C allocation, which is tracked through seven explicit plant pools: living leaves, dead standing leaves, living sapwood, heartwood, fine roots, non‐structural carbohydrates (NSC) and reproductive tissues (fruits and flowers). These pools support tissue turnover and plant respiration processes. Autotrophic respiration ($r_{\mathrm{aut},\mathrm{TC}}$; g C m^−2^ day), which encompasses maintenance and growth respiration, is represented as a process that responds to tissue‐specific metabolic demands. $r_{\mathrm{aut},\mathrm{TC}}$ utilizes C from the NSC pool when current C assimilation is insufficient to meet respiratory requirements. As such, T&C‐SMEW represents $r_{\mathrm{aut},\mathrm{TC}}$ as:

(1) $$r_{\mathrm{aut},\mathrm{TC}}=\sum \left(\mathrm{Rm}r_{H},\mathrm{Rm}r_{L},Rg_{H},Rg_{L}\right)$$

where, $\mathrm{Rm}r_{H}$ and $\mathrm{Rm}r_{L}$ (g C m^−2^) correspond to root maintenance respiration for high and low‐stature vegetation. Similarly, $Rg_{H}$ and $Rg_{L}$ (g C m^−2^) denote growth respiration for high and low‐stature vegetation. The subscript TC represents model components simulated in T&C‐BG (Fatichi et al. 2019).

Each structural C pool (leaves, living sapwood, fine roots, and carbohydrate reserves) has a target nutrient quantity necessary for construction; otherwise, nutrients can be transferred to reserve pools based on prescribed stoichiometric nutrient values (Magill et al. 2004; Zaehle et al. 2014). Alterations to plant nutrient content rely on changes to structural and reserve C pools (Friedlingstein et al. 1999) and their stoichiometry relative to N, P, K (Fatichi et al. 2019), Mg, Ca, and Si. Fine roots facilitate the uptake of mineral nutrients in plants through passive absorption following transpiration‐driven water flow or active transport that moves nutrients against concentration gradients. Plant‐mycorrhizal symbioses also contribute to nutrient uptake (Fatichi et al. 2019; Hinsinger et al. 2011; Marschner and Dell 1994).

Vegetation structure changes dynamically through plant biophysical and phenological responses to environmental conditions such as seasonality and stresses, as well as water and nutrient limitations (Buckley Paules et al. 2025; Fatichi et al. 2012; Krinner et al. 2005). In turn, vegetation growth alters both soil water uptake and nutrient dynamics.

### 2.3 Soil Biogeochemical Dynamics

#### 2.3.1 Soil Organic C

The decomposition of SOC is primarily driven by biotic processes, such as microbial activity (Schimel and Weintraub 2003). T&C‐SMEW adopts the microbially explicit approach of SOC decomposition in T&C‐BG, which represents different SOC pools, including those for enzymes, microbes, and macrofauna, reflecting the various functional roles of bacteria, saprotrophic fungi, arbuscular mycorrhiza, ectomycorrhiza and earthworms (Fatichi et al. 2019).

T&C‐SMEW groups SOC into functional pools based on partitioning criteria defined in the MEND model (Wang et al. 2013; Fatichi et al. 2019). These include particulate organic C (POC), mineral‐associated organic C (MAOC), and dissolved organic C (DOC). MAOC has a turnover rate that is potentially orders of magnitude lower than POC and, therefore, is represented separately. The proportion of POC decomposed and converted to MAOC relates to soil organic matter (SOM) and reactive surface availability (Fatichi et al. 2019). DOC is immediately available to microbes under ideal soil temperature and moisture conditions.

SOC decomposition rates depend on the size of soil C pools and extracellular enzyme abundance, which in turn depend on microbial pool size and activity (Schimel and Weintraub 2003). Microbial pools are shaped by growth dynamics (Fatichi et al. 2019), which, in turn, are regulated by DOC availability and environmental conditions such as soil temperature and moisture (Lawrence et al. 2009; Wang et al. 2013). Consequently, microbial C use efficiency (CUE) emerges as a property of enzyme production and respiration. Bacteria and saprotrophic fungi metabolise DOC, whereas mycorrhizae receive C directly from their host plant (Baskaran et al. 2017). However, only bacteria and ectomycorrhizal fungi produce extracellular enzymes necessary for SOC depolymerisation (Lindahl and Tunlid 2015). T&C‐SMEW employs an adapted version of the MEND model framework (Wang et al. 2013) to represent C fluxes among SOC fractions, using Michaelis–Menten kinetics, and characterises SOC decomposition as a function of extracellular enzyme and substrate (POC/MAOC) mass (Fatichi et al. 2019).

Furthermore, T&C‐SMEW explicitly represents macrofauna due to their role in SOC metabolisation. The macrofaunal pool is currently parameterised with respect to endogeic earthworms, as they constitute the largest macrofaunal biomass pool. Soil macrofauna only interact with below‐ground SOC and do not influence litter decomposition. Litter is produced from the turnover of plant tissue due to ageing, environmental stresses, and disturbances. Eight litter pools represent both above‐ and below‐ground litter compartments and follow a modified version of the CENTURY model (Kirschbaum and Paul 2002). As below‐ground litter corresponds to a small proportion of total SOC, litter decomposition follows linear kinetics.

Ultimately, below‐ground heterotrophic respiration (*r*_het,TC_, g C m^−2^) is computed as the sum of microbial (bacterial and fungal) ($R_{\text{microbe}}$; g C m^−2^), subsurface litter ($R_{\text{litter}}$; g C m^−2^) and macrofaunal ($R_{\mathrm{ew}}$; g C m^−2^) respiration:

(2) $$r_{\mathrm{het},\mathrm{TC}}=\sum \left(R_{\text{microbe}},R_{\text{litter}},R_{\mathrm{ew}}\right)$$

#### 2.3.2 Soil Nutrient Budgets

T&C‐SMEW simulates soil biogeochemical processes at a daily time step to match the time scale of litter input, due to uncertainties surrounding the finer‐scale temporal dynamics of nutrient cycling in the soil. Soil macro‐ and micronutrient dynamics correspond to the C:nutrient ratio of a specific donor pool, as represented by the vegetation and soil biogeochemistry modules of T&C‐BG (Fatichi et al. 2019).

T&C‐SMEW provides a mechanistic representation of the N, P, and K cycles, influencing plant functioning and productivity. The temporal dynamics of NH_4_^+^ ($\frac{d\mathrm{NH}4_{\min,\mathrm{TC}}}{\mathrm{dt}}$; g N m^−2^ day^−1^), NO_3_^−^ ($\frac{d\mathrm{NO}3_{\min,\mathrm{TC}}}{\mathrm{dt}}$; g N m^−2^ day^−1^) and PO_4_^3−^ ($\frac{d\mathrm{TPO}4_{\min,\mathrm{TC}}}{\mathrm{dt}}$; g P m^−2^ day^−1^) in soils are represented using a framework adapted from T&C‐BG (Fatichi et al. 2019). T&C‐SMEW does not include NH_4_^+^ and PO_4_^3−^ exchange processes with clay minerals due to uncertainties in their parameterisation (Cavalli et al. 2015; Yu et al. 2023). NH_4_^+^ dynamics are controlled by SOM, dissolved organic N, and microbial biomass pools (Xu et al. 2013), alongside immobilisation/mineralisation, nitrification, plant uptake, leaching, NH_3_ volatilisation, and external inputs (Equation 3). Similarly, NO_3_^−^ dynamics are influenced by immobilisation/mineralisation, nitrification, plant uptake, leaching, and denitrification (Equation 4).

(3) $$\begin{array}{cc} \frac{d\mathrm{NH}4_{\min,\mathrm{TC}}}{\mathrm{dt}}\;=\; & \left(1-f_{\mathrm{org},\mathrm{lea}}\right)\lambda_{n}\;I_{\mathrm{som},\mathrm{nit}}-\mathrm{NH}4_{\mathrm{imm}/\min}\\ & -\mathrm{NO}3_{\mathrm{flx}}-\mathrm{NH}4_{\mathrm{up}}-L_{k,\mathrm{NH}4}-N_{\mathrm{VOL}}+Ex_{N} \end{array}$$

(4) $$\frac{d\mathrm{NO}3_{\min,\mathrm{TC}}}{\mathrm{dt}}=-\mathrm{NO}3_{\mathrm{imm}/\min}+\mathrm{NO}3_{\mathrm{flx}}-\mathrm{NO}3_{\mathrm{up}}-L_{k,\mathrm{NO}3}-N_{2}$$

where, $\mathrm{NH}4_{\mathrm{imm}/\min}$ and $\mathrm{NO}3_{\mathrm{imm}/\min}$ (g N m^−2^) represents bulk soil immobilisation and mineralisation of ammonium and nitrate carried out by bacteria, saprotrophic fungi, and mycorrhizae. The term $\mathrm{NO}3_{\mathrm{flx}}$ (g N m ^−2^) is nitrification and $L_{k,\mathrm{NH}4}$ and $L_{k,\mathrm{NO}3}$ (g N m^−2^) leaching. Where $f_{\mathrm{org},\mathrm{lea}}$ is the fraction of organic leaching, while $\lambda_{N}$ is the leaching coefficient of NH_4_^+^ from litter decomposition. $I_{\mathrm{som},\mathrm{nit}}$ (g N m^−2^) is the soil organic N input due to litter decomposition. $\mathrm{NH}4_{\mathrm{up}}$ and $\mathrm{NO}3_{\mathrm{up}}$ (g N m^−2^) corresponds to the sum of active and passive plant NH_4_^+^ and NO_3_^−^ uptake from the biogeochemically active soil layer. $N_{\mathrm{VOL}}$ (g N m^−2^) represents the conversion of aqueous NH_4_^+^ to gaseous NH_3_ via volatilisation, a major pathway of N loss from agricultural soils following N fertiliser application (Bouwman et al. 2002). $Ex_{N}$ (g N m^−2^) combines exogenous N inputs from atmospheric deposition, fertilisation and biological fixation into a single term. *N*_2_ (g N m^−2^) represents denitrification.

Soil organic P dynamics are represented similarly to N dynamics (Mooshammer et al. 2014; Xu et al. 2013). Temporal inorganic P dynamics primarily follow the CENTURY model framework, which represents mineral P as an undifferentiated sum of PO_4_^3−^, HPO_4_^2−^ and H_2_PO^−^ (hereafter TPO_4_) (Parton et al. 1993; Xu et al. 2013). These dynamics are characterised by SOM inputs, immobilisation/mineralisation by soil microbes, plant uptake, leaching, primary mineral (parent material) inputs, secondary mineral precipitation and weathering inputs following feedstock dissolution.

(5) $$\begin{array}{cc} \frac{d\mathrm{TPO}4_{\min,\mathrm{TC}}}{\mathrm{dt}}\;=\; & \left(1-f_{\mathrm{org},\mathrm{lea}}\right)\lambda_{p}\;I_{\mathrm{som},\mathrm{pho}}-\mathrm{TPO}4_{b,\mathrm{imm}/\min}\\ & -\mathrm{TPO}4_{f,\mathrm{imm}/\min}-\mathrm{TPO}4_{\mathrm{AM},\mathrm{imm}/\min}-\mathrm{TPO}4_{\mathrm{EM},\mathrm{imm}/\min}\\ & -\mathrm{TPO}4_{\mathrm{up}}-L_{k,P}-P_{\sec,\mathrm{ex}}+P_{\mathrm{pri},\mathrm{ex}}+Ex_{P} \end{array}$$

where, $\mathrm{TPO}4_{\mathrm{imm}/\min}$ (g P m ^−2^) represents the immobilisation and mineralisation of mineral P by bacteria ($\mathrm{TPO}4_{b}$), saprotrophic fungi ($\mathrm{TPO}4_{f}$), arbuscular mycorrhiza ($\mathrm{TPO}4_{\mathrm{AM}}$) and ectomycorrhiza ($\mathrm{TPO}4_{\mathrm{EM}}$) (g P m^−2^). $\mathrm{TPO}4_{\mathrm{up}}$ is the sum of passive and active plant uptake of TPO4 (g P m^−2^). $L_{k,P}$ (g P m^−2^) is the leaching of TPO4. *P*_pri,ex_ (g P m^−2^) represents the conversion from primary mineral to mineral solution TPO4, while *P*_sec,ex_ (g P m^−2^) is the transformation of mineral solution to secondary TPO4. Ex_*p*_ (g P m^−2^) are the exogenous P inputs included in T&C‐BG, such as atmospheric deposition and fertilisation.

T&C‐SMEW explicitly simulates the temporal dynamics of four major nutrient elements (K, Mg, Ca, Si). The corresponding inorganic nutrient cycles include four pools: (i) elements in solution, (ii) exchangeable elements (except for Si) bound to cation exchange sites (Section 2.4), (iii) elements incorporated into secondary (non‐exchangeable) minerals and (iv) elements released from the primary dissolution of parent material (Sparks and Huang 1985). Plant uptake and leaching occur through the nutrient solution pool. The base cation solution interacts with the exchangeable pool through sorption and desorption reactions (Selim et al. 1976). Chemical and physical processes, such as primary weathering, release elements from parent material into solution. T&C‐SMEW also incorporates other components in inorganic nutrient cycles, including inputs from SOM leaching, microbial decomposition of SOM (involving fungi and bacteria), and the transformation of POC to MOC and DOC. Therefore, base cation mass balances can be expressed as (Fatichi et al. 2019):

(6) $$\begin{array}{cc} \frac{dX_{\min,\mathrm{TC}}}{\mathrm{dt}}\;=\; & \left(\mathrm{\lambda x}\right)\text{Isom},X+\frac{\mathrm{fd}\left(F_{2l}+F_{2\mathrm{cb}}+F_{2\mathrm{cf}}\;\right)+\left(F_{3b}+F_{3f}\;\right)}{{\mathrm{CX}}_{\mathrm{som}}}\\ & +X_{\min,\mathrm{rel}}-X_{\mathrm{fix},\mathrm{sol}}-X_{\mathrm{ex},\mathrm{sol},\text{SMEW}}\\ & -X_{\mathrm{up}}-L_{k,X}+{\mathrm{Ex}}_{X} \end{array}$$

where λx is the leaching fraction from litter decomposition for the cation of interest. Isom, X (g *X* m^−2^) corresponds to the input of the cation X to the SOM pool from litter decomposition. A fraction of POC (fd) can be converted to DOC. The decomposition of SOM is divided into different soil organic C components and rates of decomposition associated with enzymes produced from different microbial functional pools. For example, lignin decomposition ($F_{2l}$) is mediated by saprotrophic fungi and cellulose/hemicellulose decomposition ($F_{2\mathrm{cb}}$, $F_{2\mathrm{cf}}$) by both bacteria and saprotrophic fungi, while MAOC decomposition to DOC ($F_{3b}$, $F_{3f}$) is associated with bacteria and saprotrophic fungi. ${\mathrm{CX}}_{\mathrm{som}}$ represents the mass C: cation, *X*, ratio of SOM. The term $X_{\min,\mathrm{rel}}$ (g *X* m^−2^) corresponds to the quantity of cation, X, in the primary mineral pool, *X*_fix,sol_ (g *X* m^−2^), the non‐exchangeable (complex secondary minerals) and, $X_{\mathrm{ex},\mathrm{sol},\text{SMEW}}$ (g *X* m^−2^) in the exchangeable pool. The subscript *SMEW* represents model components simulated in SMEW (Bertagni et al. 2025). $X_{\mathrm{up}}$ (g *X* m^−2^), the plant uptake of cation X, $L_{k,X}$ (g *X* m^−2^) represents the leaching of the cation from the soil column and ${\mathrm{Ex}}_{X}$ (g *X* m^−2^) corresponds to the exogenous cation inputs from fertilisers and atmospheric deposition.

Due to the commonly incongruent nature of silicate dissolution (Crundwell 2014), which results from complex interactions between dissolved and amorphous solid species, T&C‐SMEW takes a relatively parsimonious approach to determine the amount of Si (${\mathrm{Si}}_{\min,\mathrm{TC}}$ g Si m^−2^). This approach is similar to determining the cation quantity, using estimations analogous to Equation (5), excluding the exchangeable fraction term.

### 2.4 ERW Module for Biogeochemically Active Soil Layers

T&C‐SMEW expands on the previous work of the SMEW model (Bertagni et al. 2025; Cipolla et al. 2021a, 2021b), which represents weathering dynamics in upper soil layers driven by short‐term hydrological fluctuations. The new ERW module includes weathering processes represented by SMEW (Bertagni et al. 2025), such as micronutrient (Mg, Ca, Na, K, Si) cycling, and incorporates additional processes, such as strong acid weathering. The module also emphasises the non‐linear behaviour of ERW dynamics within biogeochemically active soil layers and the influence of vegetation‐associated processes on ERW. Following the previous modelling setup in T&C‐BG and SMEW (Fatichi et al. 2019; Bertagni et al. 2025), soil biogeochemical and ERW processes are conceptualised within a vertically lumped biogeochemically active soil layer.

ERW processes, including feedstock dissolution, total soil cation and anion fluxes, inorganic C and pore water alkalinity fluxes, are simulated at 10‐min intervals to capture the influence of short‐term hydro‐(bio)geochemical fluctuations (Bertagni et al. 2025). Hourly water fluxes and daily soil biogeochemical processes fed into the ERW module are assumed to be constant within this shorter time interval. Outputs (i.e., soil pH and element concentrations) from these ERW processes are then aggregated to a daily timestep when fed back to biogeochemical dynamics.

#### 2.4.1 ERW Feedstock Weathering

Feedstock is typically composed of varying proportions of mafic minerals, which release alkalinity upon dissolution. Mineral weathering rates are governed by the thermodynamic favourability of dissolution and the available reactive surface area. Enhanced weathering rates associated with feedstock dissolution are estimated using a modification of the semi‐empirical formula by Palandri and Kharaka (2004). A linear term is introduced to account for the dependence of relative dissolution rates on soil moisture, linking the dependence to the impact of water‐exposed mineral surfaces undergoing dissolution (Cipolla et al. 2021a). Dissolution kinetic parameters were obtained from Thermoddem (2020) and USGS (2004) (Supporting Information: Section 3).

#### 2.4.2 Inorganic Carbon

ERW aims to generate and sequester inorganic C, primarily as aqueous HCO_3_^−^ (which is transported and sequestered in the ocean) and pedogenic carbonates (e.g., CaCO_3_ and MgCO_3_). Following the framework of SMEW (Bertagni et al. 2025), soil inorganic C pools within T&C‐SMEW include CO_2_ within the soil air matrix derived from soil respiration, DIC and mineral carbonate dissolution. Soil respiration is a key contributor to elevated soil air CO_2_ concentrations, making it a major factor in controlling mineral weathering rates (Banwart et al. 2009; Porporato et al. 2003). T&C‐SMEW determines soil respiration as the sum of below‐ground heterotrophic and autotrophic root respiration $(r_{\mathrm{aut},\mathrm{TC}}+r_{\mathrm{het},\mathrm{TC}}$; Sections 2.2 and 2.3.1) using the framework of T&C‐BG. Furthermore, T&C‐SMEW explicitly simulates heterotrophic respiration separately from other soil processes to capture how temperature, moisture and pH independently influence the microbial decomposition of SOC (Fatichi et al. 2019).

Since the equilibration timescale between aqueous and gaseous forms of inorganic C is shorter than mineral carbonate precipitation and dissolution, two distinct pools are defined in the ERW module: a gaseous and aqueous mineral inorganic pool (Bertagni et al. 2025). These can be combined to represent the mass balance of total inorganic C (${\mathrm{IC}}_{\mathrm{tot},\text{SMEW}}$; g C m^−2^):

(7) $$\begin{array}{cc} \frac{d{\mathrm{IC}}_{\mathrm{tot},\text{SMEW}}}{\mathrm{dt}}\;=\; & \left(r_{\mathrm{het},\mathrm{TC}}+r_{\mathrm{aut},\mathrm{TC}}\right)+I_{w}\cdot \left[{\mathrm{DIC}}_{\text{rain}}\right]\\ & -L_{k,\mathrm{IC}}-F_{\mathrm{ADV}+\text{DIFF}}+W_{\left(\mathrm{Ca},\mathrm{Mg}\right)\mathrm{CO}3} \end{array}$$

where ${\mathrm{DIC}}_{\text{rain}}$ is the concentration of DIC within rainwater (g C m ^−3^), scaled to a mass per unit volume basis, and $F_{\mathrm{ADV}+\text{DIFF}}$ (g C m^−2^) represents the advection and diffusion of gaseous CO_2_ from soil pores to the atmosphere. $L_{k,\mathrm{IC}\left[\mathrm{mob}\right]}$ pertains to leaching dissolved inorganic C (g C m ^−2^) from the biogeochemically active soil layer. Leaching of DIC and nutrients (Supporting Information: Section 2.6) from the soil is assumed to be proportional to the amount of dissolved substance and water leakage at the soil bottom, divided by the water volume in the entire soil column (Porporato et al. 2003). $W_{\left(\mathrm{Ca},\mathrm{Mg}\right)\mathrm{CO}3}$ (g C m^−2^) represents the net release (> 0) or uptake (< 0) of C resulting from the respective dissolution or precipitation of secondary carbonate minerals. $I_{w}$ (m) represents a background source of infiltrating water containing dissolved inorganic C, which can also be applied to nutrients, such as base cations, aluminium and anions.

#### 2.4.3 Magnesium, Calcium, and Potassium Balances

The dynamics of cations in soil solution (K^+^, Ca^2+^, Mg^2+^) ($\frac{dX_{\min,\mathrm{TC}}}{\mathrm{dt}}$; g *X* m^−2^ timestep^−1^) is coupled with ERW processes to determine the total quantity of the cation, *X*, in soils ($\frac{dX_{\mathrm{tot}}}{\mathrm{dt}}$; g *X* m^−2^ timestep^−1^) following feedstock application. The total soil cation quantity per time step is defined as the combined contribution of base cations in soil solution and cations released through ERW‐related processes EWx (g *X* m^−2^), which encompass soil exchange reactions alongside secondary mineral formation ($W_{\left(\mathrm{Ca},\mathrm{Mg}\right)\mathrm{CO}3}$; g *X* m^−2^):

(8) $$\frac{dX_{\mathrm{tot},\text{SMEW}}}{\mathrm{dt}}=\mathrm{EW}x+W_{\left(\mathrm{Ca},\mathrm{Mg}\right)\mathrm{CO}3}+\frac{dX_{\min,\mathrm{TC}}}{\mathrm{dt}}$$

#### 2.4.4 Ammonium, Nitrate, and Phosphate Balances

Acidifying processes indirectly influence soil alkalinity and ERW processes, such as atmospheric N deposition and N fertiliser application, which enhance nitrification (Wang et al. 2020), as well as P fertiliser inputs and P contributions from feedstock dissolution. T&C‐SMEW incorporates NH_4_^+^, NO_3_^−^ and TPO_4_ dynamics ($\frac{d\mathrm{NH}4_{\min,\mathrm{TC}}}{\mathrm{dt}}$, $\frac{d\mathrm{NO}3_{\min,\mathrm{TC}}}{\mathrm{dt}}$, $\frac{d\mathrm{TPO}4_{\min,\mathrm{TC}}}{\mathrm{dt}}$; g *X* m^−2^ day^−1^, described in Section 2.3.2) with ERW processes to quantify their respective capacities to shift carbonate system equilibria and modify weathering dynamics.

Some feedstocks contain minerals such as apatite, which consists of small quantities of P (Lewis et al. 2021). This additional P source is represented in T&C‐SMEW as EWp (g P m ^−2^). Therefore, the total amount of soil P ($\frac{\mathrm{PO}4_{\mathrm{tot},\text{SMEW}}}{\mathrm{dt}}$; g P m^−2^ timestep^−1^) is determined as the sum of feedstock‐derived P and soil mineral P:

(9) $$\frac{\mathrm{dPO}4_{\mathrm{tot},\text{SMEW}}}{\mathrm{dt}}=\mathrm{EW}p+\frac{\mathrm{dTPO}4_{\min,\mathrm{TC}}}{\mathrm{dt}}$$

#### 2.4.5 Sodium, Aluminium, and Silicate Balances

Al and Na can impact the absorption of other nutrients by increasing plant stress (Ma et al. 2022; Ofoe et al. 2023; Zhu 2016). The influence of Al on the weathering process is primarily evident at low pH values (< 5.0), which are not conducive to ERW‐related CDR (Bertagni and Porporato 2022; Rengel et al. 2015; Ur Rahman et al. 2024), and given that Al uptake by plants is minimal at low soil pH, due to its toxicity (Ofoe et al. 2023), Al uptake is not represented. By contrast, Na uptake is assumed to occur passively (${\mathrm{Na}}_{\mathrm{up},p,\mathrm{TC}}$; g Na m^−2^). Therefore, the mass balances to determine the total soil amount of Al (${\mathrm{Al}}_{\mathrm{tot},\text{SMEW}}$; g Al m^−2^) and Na (${\mathrm{Na}}_{\mathrm{tot},\text{SMEW}}$; g Na m^−2^), are represented as:

(10) $$\frac{d{\mathrm{Al}}_{\mathrm{tot},\text{SMEW}}}{\mathrm{dt}}=I_{\mathrm{Al},\mathrm{TC}}+EW_{\mathrm{Al}}-L_{k,\mathrm{Al}\left[\mathrm{mob}\right],\mathrm{TC}}$$

(11) $$\frac{d{\mathrm{Na}}_{\mathrm{tot},\text{SMEW}}}{\mathrm{dt}}=I_{\mathrm{Na},\mathrm{TC}}+EW_{\mathrm{Na}}-L_{k,\mathrm{Na},\mathrm{TC}}-{\mathrm{Na}}_{\mathrm{up},p,\mathrm{TC}}$$

where $I_{\mathrm{Na},\mathrm{TC}}$ (g Na m ^−2^) and $I_{\mathrm{Al},\mathrm{TC}}$ (g Al m ^−2^) are bulk external input terms stemming from processes such as atmospheric deposition, while $EW_{\mathrm{Na}}$ (g Na m ^−2^) and $EW_{\mathrm{Al}}$ (g Al m^−2^) correspond to the respective release of Na and Al from feedstock weathering. $L_{k,\mathrm{Al}\left[\mathrm{mob}\right],\mathrm{TC}}$ (g Al m ^−2^) and $L_{k,\mathrm{Na},\mathrm{TC}}$ (g Al m^−2^) relates to the export of mobile Al and Na in soil pore water.

The soil solution Si mass balance is determined using the framework of T&C‐BG (Section 2.1.3). However, the total Si mass balance is determined by summing the Si in soil ($\frac{d{\mathrm{Si}}_{\min,\mathrm{TC}}}{\mathrm{dt}}$; g Si m^−2^ day^−1^) and derived from enhanced weathering of feedstock (${\mathrm{EW}}_{\mathrm{Si}}$; g Si m^−2^):

(12) $$\frac{d{\mathrm{Si}}_{\mathrm{tot},\text{SMEW}}}{\mathrm{dt}}={\mathrm{EW}}_{\mathrm{Si}}+\frac{d{\mathrm{Si}}_{\min,\mathrm{TC}}}{\mathrm{dt}}$$

where ${\mathrm{Si}}_{\mathrm{tot},\text{SMEW}}$ (g Si m ^−2^) corresponds to the total Si content within a volume of the biogeochemically active soil layer and is linked to ERW dynamics with the input term, ${\mathrm{EW}}_{\mathrm{Si}}$.

#### 2.4.6 Residual Anion Balance

Another mass balance approach accounts for strong anions (e.g., Cl^−^ and SO_4_^2−^) not explicitly represented in individual mass balance equations. These are incorporated into a generic anion pool, as the scale of their inputs is smaller than that of N and P. Although these inputs are minor in quantity, they have a significant influence on biogeochemical processes, such as soil respiration (Zheng et al. 2023), and have been substantially affected by anthropogenic activities in recent decades (Haskins et al. 2020).

Owing to uncertainties surrounding the uptake of anions such as Cl^−^ and SO_4_^2−^ (Reich et al. 2017), this process is represented using passive plant uptake of a lumped pool (${\text{An}}_{\mathrm{up},p,\mathrm{TC}}$; g An m ^−2^). Moreover, given the conservative nature of alkalinity export (Wolf‐Gladrow et al. 2007) and the minimal impact of anion immobilisation in soils (Bertagni et al. 2025), the dynamics of other major anions (${\text{An}}_{r,\mathrm{tot},\text{SMEW}}$; g An m^−2^) in soil pore water are determined within the ERW module. This process is represented using a residual anion pool and is influenced by leaching and passive uptake:

(13) $$\frac{d{\text{An}}_{r,\mathrm{tot},\text{SMEW}}}{\mathrm{dt}}=I_{\text{An},\mathrm{TC}}-L_{k,\text{An},\mathrm{TC}}-{\text{An}}_{\mathrm{up},p,\mathrm{TC}}$$

#### 2.4.7 Soil Pore Water Alkalinity

By incorporating the pore water alkalinity framework of SMEW (Bertagni et al. 2025), an explicit equation for alkalinity $\left[\mathrm{Alk}\right]$ (mol m^−3^) is defined as the charge balance between major cations and anions and can be expressed through the sum of proton donors minus acceptors (Wolf‐Gladrow et al. 2007). This indirectly links alkalinity to the aqueous carbonate buffering system, as (bi)carbonate acts as a major proton acceptor, buffering the effects of acidity. A detailed definition of alkalinity, as used in T&C‐SMEW, is provided in Supporting Information: Section 2.7.

(14) $$\begin{array}{cc} \left[\mathrm{ALK}\right]= & 2\left[{\mathrm{Ca}}^{2+}\right]+2\left[{\mathrm{Mg}}^{2+}\right]+\left[K^{+}\right]+\left[{\mathrm{Na}}^{+}\right]\\ & +\left[{\mathrm{NH}}_{4}^{+}\right]-\left[{\mathrm{NO}}_{3}^{-}\right]-\left[{\text{An}}_{r}\right]-\left[{\mathrm{TPO}}_{4}\right] \end{array}$$

In which $\left[{\mathrm{Mg}}^{2+}\right]$, $\;\left[{\mathrm{Ca}}^{2+}\right]$, $\left[K^{+}\right]$, $\left[{\mathrm{Na}}^{+}\right]$, $\left[{\mathrm{NH}}_{4}^{+}\right]$ (mol m ^−3^) indicates concentrations of major cations. $\left[{\text{An}}_{r}\right]$ (mol m ^−3^) is the concentration of anions within the residual pool, while $\left[\mathrm{NO}3^{-}\right]$ (mol m ^−3^) corresponds to the nitrate concentrations. $\left[{\mathrm{TPO}}_{4}\right]$ represents the undifferentiated sum of PO_4_^3−^, HPO_4_^2−^ and H_2_PO^−^, as outlined in Section 2.3.2.

T&C‐SMEW estimates alkalinity from the concentrations of cations and anions (Equation 14) and predicts pH through equilibrium reactions, accounting for temporal effects of plant nutrient uptake on alkalinity and pH (Banwart et al. 2009; Bolan et al. 1991; Kelly et al. 1998).

## 3 Model Setup

T&C‐SMEW requires a similar setup to SMEW (Bertagni et al. 2025) and T&C‐BG (Fatichi et al. 2019) to compare model outputs against experimental observations (e.g., Section 5). Model inputs include hourly meteorological forcings (air temperature, dew point temperature, precipitation, relative humidity, atmospheric pressure, wind speed, downwelling short and longwave radiation), daily atmospheric CO_2_ concentrations, vegetation traits, soil texture, initial conditions of soil biogeochemistry pools, ERW material, application schedule and rate. Following SMEW, a dissolution factor (*F*_D_) was introduced to modify the weathering rate formulation of Palandri and Kharaka (2004), accounting for uncharacterised biotic and abiotic processes that affect feedstock dissolution rates. It represents the enhancement (*F*_D_ > 1) or inhibition (*F*_D_ < 1) of mineral weathering. All meteorological forcings and parameters were set to align with the experimental conditions and published measurements; otherwise, additional datasets and estimations were used, as listed in Supporting Information: Section 3.

Hourly meteorological forcings were obtained from ERA5‐Land (Muñoz Sabater 2019). Atmospheric CO_2_ concentrations followed historical observations during the experimental periods (Monroe 2025). Vegetation parameters were derived from detailed field observations where available and otherwise adapted from datasets corresponding to defined plant functional types (Paschalis et al. 2024, 2025), aiming to capture comparable vegetation dynamics despite limited site‐specific data and to ensure the broad applicability of these functional classifications (Supporting Information: Section 3).

Biogeochemical model parameters were adopted from the default values as listed in Fatichi et al. (2019), given the limited knowledge available. Some of these parameters are likely case‐study specific, reflecting the complexity of soil microbial community dynamics and their coupled interactions with nutrient cycling processes, which may lead to uncertainty in modelling results. However, a full sensitivity analysis is beyond the scope of this study. Instead, we discuss the implications of applying default parameter sets and associated uncertainties in a field setup in Section 5.1.

The initial fraction of base cations adsorbed to soil exchange sites and the initial concentrations of base cations in the soil solution were obtained through study observations. If no data were available, the soil biogeochemistry module was initialised with a spin‐up procedure to determine base cations in the soil solution and exchangeable pool. A detailed spin‐up procedure is provided in Supporting Information: Section 5.

## 4 Model Comparison With Experimental Observations

Model simulations were compared against two experiments to assess the accuracy of T&C‐SMEW: (a) a mesocosm study involving sorghum establishment and growth across 121 days (Kelland et al. 2020), as investigated by Bertagni et al. (2025) and (b) a four‐year field experiment involving a maize‐soy rotation in Illinois, USA (Beerling et al. 2024; Kantola et al. 2023). The latter experiment utilises the ability of T&C‐SMEW to run field‐scale simulations, allowing us to compare model outputs against observed long‐term dynamics following silicate rock amendment. Parameter sets used for each experiment are listed in Supporting Information: Sections 3 and 4, along with the accompanying model parameter files.

Because the mesocosm study was conducted under controlled conditions, parameter uncertainty for weathering processes was comparatively low. The basalt feedstock was well‐characterised (Table 1 in Kelland et al. (2020)) with dissolution kinetics determined under similar conditions to the mesocosm experiment, reducing uncertainty in the representation of feedstock weathering rates. The simplified system, including a single crop ( *Sorghum bicolor* ), grown in uniform columns (refer to Section 4.1 and Supporting Information: Section 3 for more details), avoided the complexity of mixed vegetation, variable rooting depths and soil heterogeneity that complicate field studies. Observational measurements were also conducted over a short time span, resulting in lower temporal uncertainty. As a result, an uncertainty analysis was restricted to the more complex field experiment, where greater variability in soil heterogeneity, hydrological dynamics and measurement error required explicit quantification of uncertainty.

### 4.1 Mesocosm With Sorghum

The 121‐day basalt weathering experiment was conducted within cylindrical plastic columns containing the C4 crop, *Sorghum bicolor* , which was grown under controlled laboratory conditions (Kelland et al. 2020). The detailed model setup and relevant key parameters are summarised in Supporting Information: Section 3.1 and Table S1. The dissolution factor, *F*_D_, for Oregon basalt (Lewis et al. 2021), used in this mesocosm experiment, was adjusted to 0.5 to reproduce the reported alkalinity release (i.e., potential CDR).

#### 4.1.1 Model‐Data Comparison

**FIGURE 2:**
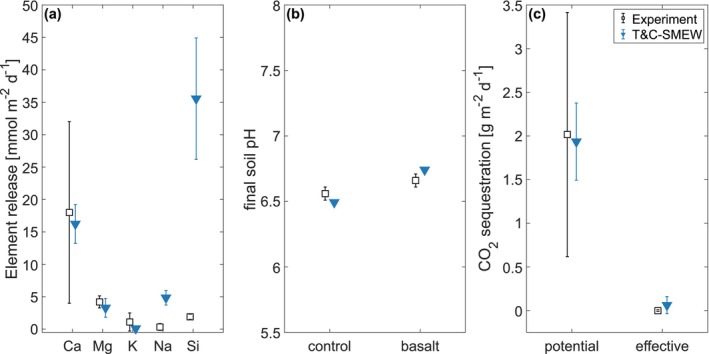
Comparisons between the mesocosm experiment (black) by Kelland et al. (2020) and the T&C‐SMEW model simulation (blue). (a) Average daily basalt dissolution releases the elements Ca, Mg, K, Na, and Si, expressed per land surface unit (mmol m^−2^ d^−1^). (b) Soil pH after 120‐day experiment. (c) Daily average potential (alkalinity release) and effective (aqueous carbonate leaching) CO_2_ sequestration (g m^−2^ d^−1^). Error bars represent ± one standard deviation calculated from replicate measurements in the experiment and model estimates. Comparisons with SMEW (Bertagni et al. 2025) are presented in Table S4.

The coupled TC‐SMEW model represents the release of Ca, Mg and K from feedstock and associated CO_2_ sequestration, with all estimates falling within the range of observational errors (Figure 2a,c and Table S4). This highlights the ability of T&C‐SMEW to represent soil biogeochemical processes and their interactions with the aqueous‐carbonate system. Additionally, the simulated final soil pH aligns with observations, demonstrating a modest reduction in soil acidity following basalt application (Figure 2b and Table S4).

**FIGURE 3:**
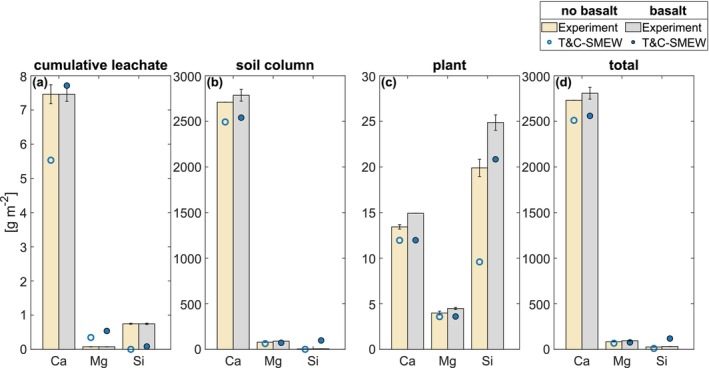
Comparisons of Ca, Mg and Si budget (g m^−2^) partitioning in (a) leachate, (b) soil, (c) plant and (d) total (whole ecosystem: cumulative leachate, soil column and plant), expressed per unit surface land, in basalt‐amended and unamended treatment groups. Bars represent mean values, while error bars display ± one standard deviation among all experimental measurements. Comparisons with SMEW (Bertagni et al. 2025) are presented in Table S5.

The magnitude of simulated Ca and Mg partitioned among the leachate, soil and plant pools closely aligns with observations (Figure 3 and Table S5), demonstrating the capacity of the model to represent feedstock dissolution and the partitioning of major cations into different ecosystem components. The discrepancy exists in simulated Ca and Mg concentrations in plant tissues, where the simulation displays a muted response to feedstock addition, as the reserve nutrient pools were already saturated before the feedstock was applied (Figure 3c and Table S5).

The model overestimates the cumulative leachate response following silicate rock amendment (Figure 3a), as it simplifies element leaching dynamics by treating them as proportional to total soil column contents. Nevertheless, the magnitude of leachate partitioning remains small across component budgets in both observations and simulations, leading to a limited impact on overall ecosystem dynamics.

The model overestimates Si release from feedstock dissolution (Figure 2a), leading to higher simulated Si contents in the soil column compared to measurements (Figure 3b). However, consistent with observations, the simulated Si concentrations in plant tissues also increase following the addition of silicate rock (Figure 3c). Although the magnitudes are underestimated, this likely reflects model uncertainty in the prescribed plant stoichiometric ratio.

The tendency of the model to overestimate Na and Si release, together with its difficulty in representing Si partitioning, may reflect the inherent complexity of simulating incongruent Si release from the feedstock. Biota likely drove weathering reactions, resulting in incongruent mineral dissolution (Thorley et al. 2014). In addition, complex, slow‐weathering secondary clay minerals, such as smectite in the feedstock (Lewis et al. 2021), may have acted as an additional sink, reducing feedstock‐water contact and limiting the observed Si release.

### 4.2 A Field Experiment With a Maize‐Soy Rotation

The second model‐data comparison involves an agricultural field study conducted in Illinois, USA (Kantola et al. 2023; Beerling et al. 2024). This study applied 4 rounds of a comprehensively characterised Blue Ridge basalt, at a rate of 5 kg m^−2^, to plots with a maize‐soybean ( *Zea mays* ‐ *Glycine max* ) rotation from November 2016 until 2020. The detailed model setup and relevant key parameters are summarised in Supporting Information: Section 3.2 and Table S1.

The Blue Ridge feedstock contained a high proportion of medium‐weathering silicate materials such as plagioclase; however, its mineralogy was reported by Lewis et al. (2021) and Beerling et al. (2024) with different results from XRD analysis. Lewis et al. (2021) used scanning electron microscope‐energy dispersive X‐ray spectroscopy (SEM‐EDS) spectra to semi‐quantitatively compare the mineral elemental composition using the WebMineral Element Composition Search (WebMineral 2025). A large elemental measurement tolerance was required to identify measured elemental weight percentages for known minerals. To account for uncertainty in characterising feedstock mineralogy and elemental composition (Möller and Dupla 2025), mineral assemblages were adopted and represented individually as: 19.6% albite, 11.6% ferroactinolite, 25.6% epidote, 36.3% chlorite and 5.2% quartz for Lewis et al. (2021) and 23.3% albite, 11.9% ferroactinolite, 17.8% epidote applied here as a proxy for the reported piemontite, due to limited information of its dissolution parameters (piemontite belongs to the epidote endmember group), 34% chlorite, 9% quartz and 2.6% calcite for Beerling et al. (2024). Herein, we refer to basalt characterised by Lewis et al. (2021) as basalt_L_ and that which was characterised by Beerling et al. (2024) as basalt_B_. Based on the reported mineralogy in both studies, no P source was released from the feedstock. An *F*_D_ value of 1 was applied for both mineralogy simulations (i.e., no correction to the theoretical weathering rates of Palandri and Kharaka (2004)).

Some model parameters were insufficiently informed by available data. Therefore, they were assigned default values from prior applications (Fatichi et al. 2019), while parameter and variable uncertainty ranges were systematically defined to encompass plausible variability in hydrology (effective soil pore saturation), soil biogeochemistry (e.g., maximum specific decomposition rate for MAOC), and weathering dynamics (e.g., *F*_D_) under field conditions. A set of simplified Monte Carlo simulations (conducted using maximum, minimum and original values for each selected parameter; hereafter denoted with the subscript uc) captured the uncertainty in modelled CDR (Figure 7), as well as a potentially low signal‐to‐noise field measurement. The detailed uncertainty simulation setup is summarised in Supporting Information: Section 4.

#### 4.2.1 Field Observation‐Model Comparison

**FIGURE 4:**
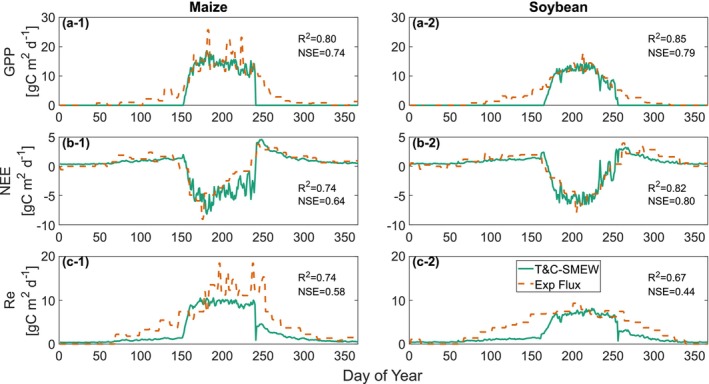
Annual time series of average daily ecosystem carbon flux, partitioned into (a) gross primary production (GPP), (b) net ecosystem exchange (NEE), and (c) ecosystem respiration (Re) (g m^−2^ d^−1^) during the pretreatment period (2009–2016) across the maize (a‐1, b‐1, c‐1) and soybean (a‐2, b‐2, c‐2) rotations (Kantola et al. 2023). Solid green lines represent simulated values, while dotted lines denote observations extracted from the AmeriFlux network.

For the maize and soybean rotations, T&C‐SMEW demonstrates reasonable agreement with observed temporal ecosystem carbon dynamics during pretreatment years, supporting the suitability of model vegetation parameters: GPP (maize: *R* ^2^ = 0.80, NSE = 0.74; soybean: *R* ^2^ = 0.85, NSE = 0.79; Figure 4a‐1,a‐2), NEE (maize: *R* ^2^ = 0.74, NSE = 0.64; soybean: *R* ^2^ = 0.82, NSE = 0.80; Figure 4b‐1,b‐2) and ecosystem respiration (Re; maize: *R* ^2^ = 0.74, NSE = 0.58; soybean: *R* ^2^ = 0.67, NSE = 0.44; Figure 4c‐1,c‐2).

**FIGURE 5:**
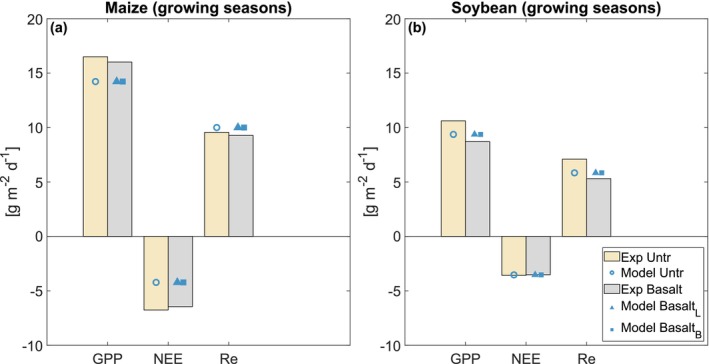
Mean ecosystem carbon flux partitioned into gross primary production (GPP), net ecosystem exchange (NEE) and ecosystem respiration (Re) (g m^−2^), separated by (a) maize (2017, 2018, 2020) and (b) soybean (2019) rotations and integrated across growing seasons, respectively, during treatment years. Grey bars represent mean experimental values from basalt treatments, and orange bars from control/untreated treatments (Kantola et al. 2023). Blue triangular/square points correspond to simulated mean values using the Lewis et al. (2021)/Beerling et al. (2024) feedstock characterisation. Similarly, the subscripts L and B relate to feedstock mineralogy from Lewis et al. (2021) and Beerling et al. (2024), respectively.

During treatment years, with the same set of vegetation parameters applied, the model still captures the trend of ecosystem fluxes (Figures S3 and S4), especially during the crop‐growing seasons. When ecosystem fluxes are integrated across maize and soybean rotation growing seasons, the model aligns with the magnitude of each flux (Figure 5 and Table S6).

**FIGURE 6:**
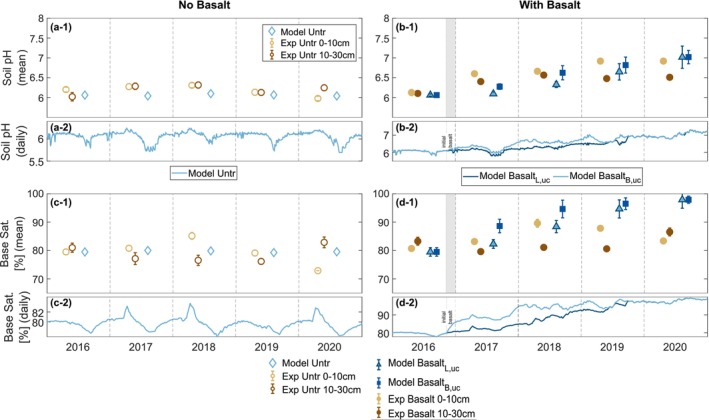
Mean annual and daily (a, b) soil pH and (c, d) base saturation (%) in (a, c) control (untreated/no basalt) and (b, d) basalt amended plots. T&C‐SMEW simulates soil pH at a 10‐min resolution within a lumped active layer of the entire 30 cm soil profile (error bars are ± one standard deviation in uncertainty simulations), whereas measurements were taken at discrete time points and sampling depths (i.e., 0–10 cm and 10–30 cm; error bars are ± standard error of the mean). Hollow scatter points: no basalt; solid scatter points: with basalt; yellow/brown: Experimental observations from the upper 10 cm/the 10–30 cm soil layer; blue. Modelling results; triangular/square: Simulated values using the Lewis et al. (2021)/Beerling et al. (2024) feedstock characterisations. The shaded regions in sub‐plots b1 and c1 highlight the period of basalt application in November 2016, following the pretreatment period. Similarly, the subscripts L and B relate to feedstock mineralogy from Lewis et al. (2021) and Beerling et al. (2024), respectively.

T&C‐SMEW simulates soil pH at a 10‐min resolution within a vertically lumped biogeochemically active soil layer, 30 cm in depth (Figure 6a,b), while experimental measurements were taken at discrete timepoints and sampling depths (0–10 cm and 10–30 cm; Figure 6a,b, Table S7; Beerling et al. 2024). In the control case, the simulated annual average pH remains stable at approximately 6.1 over the 4‐year period, aligning reasonably with the variability observed across the measured depths. However, the simulation reveals minor seasonal fluctuations in soil pH resulting from variations in soil moisture and crop growth dynamics (Figure 6a‐2).

In the basalt treatment, with both feedstock mineralogies (Lewis et al. 2021; Beerling et al. 2024), the simulated soil pH consistently increases, aligning with observations. In 2018, basalt_B_ raised the soil pH by 0.3 units more compared to basalt_L_. However, by 2020, both mineralogies converged to a pH of 7.0 (Table S7). This source of uncertainty emphasises the importance of well‐characterised feedstock mineralogy in modelling studies.

In addition, the system response to basalt_B_ and basalt_L_ application displays a one‐year lag before a discernible increase in simulated soil pH in the model. This delay may be due to a slower dissolution rate simulated by the model or an increased pH that was not well represented in the control case during 2017. The lag may also reflect the time required for the progressive saturation of cation exchange sites before alkalinity accumulates to levels sufficient for detectable increases in soil pH.

**FIGURE 7:**
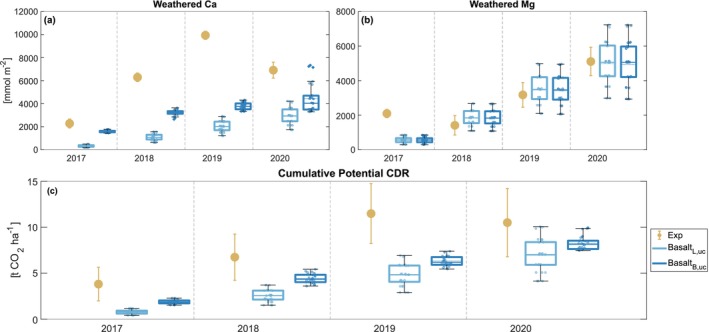
Mean annual cumulative dissolved (weathered) (a) Ca and (b) Mg, as well as (c) cumulative potential carbon dioxide removal (CDR), represented as the sum of dissolved Ca and Mg in bulk soil. Yellow points represent experimentally observed values. Triangular/square points correspond to simulated values using the Lewis et al. (2021)/Beerling et al. (2024) feedstock characterisations. Subscripts L, UC and B, UC relate to uncertainty analysis simulations using feedstock mineralogy from Lewis et al. (2021) and Beerling et al. (2024), respectively. In experimental measurement plots, error bars represent ± standard error of the mean. The box plot illustrates the spread of simulated estimates derived from a plausible range of model inputs and parameter values related to weathering, vegetation and soil biogeochemistry.

Model simulations reflect an increasing response in experimental observations of weathered Ca and Mg following annual feedstock applications, irrespective of the feedstock characterisation used to initialise model simulations (Figure 7a,b and Table S8). Consistent with varying modelled pH responses, basalt_B,uc_ displays greater Ca release and cumulative potential CDR (the sum of weathered Mg and Ca concentrations) than basalt_L,uc_, especially in 2018 and 2019. While the modelled and observed weathered Mg is largely consistent, the modelled Ca was underestimated, likely due to the slower simulated weathering rate of Ca‐rich minerals (e.g., the epidote endmember group mineral, Figure S5). Consequently, the mean modelled CDR was underestimated, although the estimated uncertainty for basalt_B_ (displayed by the dark blue box plots) generally falls within the experimental measurement uncertainty range, except for 2019.

**FIGURE 8:**
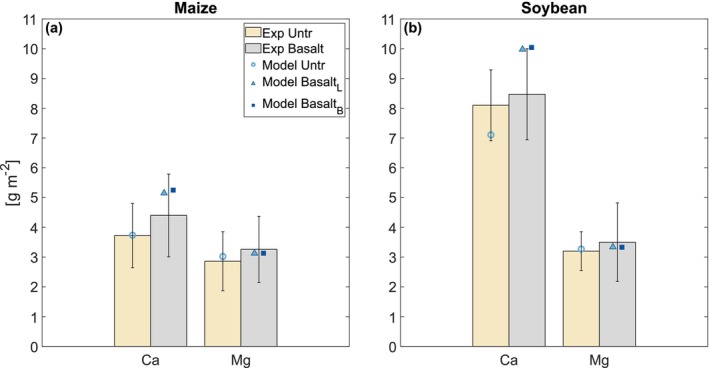
(a) Maize and (b) soybean (leaf, root and stem) tissue Ca and Mg concentrations (g m^−2^) averaged across experimental years, 2017–2020. Grey bars represent mean experimental values from basalt treatments, and orange bars from control/no basalt treatments (Kantola et al. 2023). Error bars represent standard errors from experiment measurements among all plots. Triangular points correspond to simulated values using the Lewis et al. (2021) feedstock characterisation, and square points to the Beerling et al. (2024) characterisation. The subscripts L and B relate to feedstock mineralogy from Lewis et al. (2021) and Beerling et al. (2024), respectively.

Across maize and soybean rotations, the model reliably represents plant tissue Ca and Mg concentrations in both basalt and no‐basalt treatments (Figure 8 and Table S9). Following feedstock amendment, irrespective of mineralogy, soybean uptakes more Ca relative to Mg compared to maize (Figure 8 and Table S9), highlighting the ability of the model to represent plant functional type‐dependent nutrient uptake.

## 5 Discussion

T&C‐SMEW explicitly represents microbial processes (Fatichi et al. 2019) and ERW dynamics (Bertagni et al. 2025) through the detailed representation of hydroclimatic variability and coupled biotic‐abiotic interactions driving weathering processes. The resulting modelling framework provides mechanistic insights into the roles of soil moisture, biotically mediated SOC decomposition, plant nutrient uptake, respiration, soil physicochemistry and feedstock dissolution in alkalinity production and export from the soil column, and their corresponding influence on ERW‐induced CDR.

T&C‐SMEW simulations were compared against a controlled mesocosm (Kelland et al. 2020) and a long‐term field study (Kantola et al. 2023; Beerling et al. 2024). The mesocosm study offered insights into hydrologic constraints and vegetation effects on ERW processes, while the field study demonstrated the real‐world impacts of CDR, including changes in soil respiration, primary production and alkalinity export.

### 5.1 Model Evaluation

#### 5.1.1 Modelled Ecosystem C Dynamics and Associated Uncertainty

The model captures the reported daily ecosystem C budget from the field experiment, particularly for NEE, which was measured directly using eddy covariance towers. Mismatches are more evident in the other two ecosystem C budgets (Re and GPP), especially during non‐growing seasons. The difference between observed and simulated Re in winter and spring months (Figure 4c‐1,c‐2) likely arises from the method used to reconstruct Re from NEE, which relies on the nighttime temperature response function of Lloyd and Taylor (1994) and introduces a source of estimate uncertainty. In addition, edge effects associated with photosynthetic activity at nearby sites, detected by eddy covariance flux towers, may contribute to discrepancies between Re and GPP during spring and summer (Figure 4a‐1,c‐1,a‐2,c‐2).

Such uncertainty in observational measurements poses a major challenge for robust model comparisons, complicating the distinction between measurement error and genuine model–observation mismatches. Since different methods were used to estimate various components of the ecosystem C budget, such as eddy covariance towers (for NEE), soil respiration measurements (partitioned into autotrophic (root) and heterotrophic components) and biomass sampling (Kantola et al. 2023), the resulting observational budget of various components is not balanced and should be considered an approximation.

Further model‐observation variation may be related to the partitioning of soil respiration into heterotrophic and autotrophic components (Figure S2). Kantola et al. (2023) report that overall changes in soil respiration following feedstock amendment were minor. This suggests that part of the variation may be driven by secondary biomass effects not captured by the model. For example, enhanced root growth under a no‐till management regime can increase root respiration and nutrient‐related processes, such as the exudation of organic acids that influence microbial respiration (de Vries et al. 2019; Hou et al. 2025).

#### 5.1.2 Modelled Vegetation Dynamics and Associated Uncertainty

Simulated crop growth dynamics align with the expectation that plant uptake enhances mineral weathering rates by decreasing saturation of weathering products in pore waters, alleviating a thermodynamic constraint on feedstock dissolution (Figure 6) (Amann et al. 2020; Buckingham and Henderson 2023). This alleviation can be limited when plant nutrient reserves saturate, slowing uptake rates even when additional elements are released from the feedstock. The model captures this behaviour in both field and mesocosm experiments, particularly in plant Mg budget response following silicate rock amendment. Within T&C‐SMEW, each plant tissue has a specific nutrient quantity required for its construction. The model implements stoichiometric flexibility, increasing nutrient concentrations to 80% of the maximum storage capacity before uptake is suppressed (Fatichi et al. 2019). Consequently, even under high feedstock dissolution rates, plant uptake of specific cations may not change significantly if the structural tissue approaches saturation with respect to a specific cation, since further uptake becomes progressively limited by this regulatory mechanism.

However, a source of uncertainty in the model arises from the limited availability of data on plant tissue elemental concentrations and reserve saturation, particularly for Si. The underestimation of Si concentration in C4 plants (Figure 3c) is likely due to unrepresentative model parameterisation, while the saturation of Ca reserves explains the underestimated plant tissue Ca observed in soybeans (Figure 8b). Expanding plant tissue elemental datasets can limit model uncertainty in simulating plant uptake and ecosystem responses to ERW.

Additionally, Pavlovic et al. (2021) suggest Si has variable effects on Ca uptake, potentially reducing tissue Ca concentrations under periods of plant stress and negatively impacting plant growth and GPP. The discrepancy between the observed and simulated feedstock Si release (Figure 2a) suggests that the model may also underrepresent this process, potentially impacting modelled Si and Ca plant uptake (Figure 3c). Future model iterations could improve plant nutrient dynamics by enhancing parameterisation and Si availability‐dependent tissue stoichiometry, thereby enabling greater tissue nutrient flexibility and better capturing the effects of feedstock amendment and plant nutrient uptake on GPP (Etesami and Jeong 2018), as well as Re and NEE.

#### 5.1.3 Modelled Soil Physicochemical Dynamics and Associated Uncertainty

The simulated increase in soil pore water pH, here used as a proxy of bulk soil pH, aligns with experimental observations (determined with a 1:1 soil: H_2_O ratio following Carter and Gregorich (2007)). Although the absence of explicit bulk soil pH dynamics limits direct comparison, recent modelling efforts demonstrate that the relationship between bulk soil and porewater pH can be systematically approximated (Kanzaki et al. 2024). Therefore, while methodological differences should be acknowledged, approximate comparisons between bulk soil and porewater pH can still yield meaningful insights into ERW‐related geochemical processes.

The variability in simulated pH dynamics not only highlights the ability of the model to capture the interactions between soil pH and plant dynamics (displayed by the error bars in Figure 6a,b), but also the importance of reporting experimental sampling times to enable direct temporal comparisons (Figure 6a,b). Incorporating high‐frequency sampling would improve the evaluation of model performance and enhance understanding of how interconnected factors, such as vegetation, soil moisture and pH, drive weathering reactions (Jones et al. 2025).

Soil base saturation is another major factor controlling the retention and export of weathering products throughout the soil column. The model slightly overestimates base saturation in the field‐model comparison (Figure 6c‐1), likely because it does not represent NH_4_^+^ exchange processes associated with soil clay minerals. Incorporation of exchangeable NH_4_^+^ would introduce competition with base cations for exchange sites on clay minerals, thereby influencing base saturation and the base cation sorption onto exchange sites, ultimately affecting alkalinity export.

#### 5.1.4 Modelled Weathering Dynamics, CDR and Associated Uncertainty

The match between experimental and simulated weathered Ca/Mg and potential CDR values (Figures 2c and 7c) can be attributed to the ability of T&C‐SMEW to determine soil water and element dynamics. This is particularly notable in the field setup when no correction was applied to weathering dynamics (*F*_D_ = 1).

Although the framework captures dominant ecohydrological and biogeochemical controls, projected potential CDR remains sensitive to the parameterisation of mineralogical composition and dissolution kinetics. This can be demonstrated by comparing basalt_L_ and basalt_B_ and the range of their plausible impacts on feedstock cation release and CDR (displayed by the blue box plots in Figure 7). T&C‐SMEW reproduces similar mean weathered Mg in both scenarios, but exhibits a different response in weathered Ca and, therefore, simulated CDR.

The relatively slow weathering rate of epidote introduces uncertainty in Ca release (Figure S5), with consequences for alkalinity export and plant nutrient supply. For example, in the basalt_B_ measurements, the feedstock was defined as containing piemontite, a member of the epidote endmember group. However, due to the absence of a dedicated chemical formula and kinetic parameters for piemontite, we followed Lewis et al. (2021) in adopting epidote and its kinetic rate constants as a proxy. This substitution likely contributes to the underestimation of simulated weathered Ca from the epidote endmember group.

Constraining these uncertainties will require targeted laboratory and field measurements to better characterise the dissolution behaviour of key minerals under realistic soil conditions. Incorporating such constraints into future model uncertainty analyses would enhance the robustness of field‐scale CDR estimates and reduce the likelihood of systematic under‐ or overestimation of ERW effectiveness for CDR.

### 5.2 Outlook and Broader Implications

The current complexity of the biogeochemistry module derived from T&C‐BG versus the parsimonious nature of the coupled ERW module reflects the balance between process understanding and scalability, which is key for implementing ERW as a viable CDR strategy. By emphasising whole‐ecosystem responses, such as plant‐mediated cation uptake and corresponding biomass accumulation via a fertilisation effect (Kantola et al. 2023), as well as hydrologically integrated weathering fluxes, this modelling framework can be used to build numerical experiments that inform land management and policy decisions across different ecosystems and hydroclimatic regimes. The framework mechanistically captures vegetation‐mediated controls on weathering dynamics, including plant‐driven cation uptake and its associated impacts on alkalinity export and soil pH. T&C‐SMEW captures management practices, such as grazing and mowing, thereby supporting the extrapolation of ERW outcomes in agricultural and reforestation settings.

Through these capabilities, T&C‐SMEW acts as a critical tool for evaluating the CDR potential of ERW. For example, the maximum annual simulated field‐scale CDR (Figure 7c, basalt_L_ 6.9 ± 1.8 t CO_2_ ha^−1^ and basalt_B_ 8.3 ± 0.7 t CO_2_ ha^−1^ in 2020) demonstrates the substantial CDR potential of ERW. The magnitude is similar to that of re/afforestation (Griscom et al. 2017), positioning ERW as a CDR strategy with comparable effectiveness to a widely deployed nature‐based climate solution. ERW can also be co‐deployed with other CDR and land uses, reducing competition with food production and offering a unique advantage over CDR strategies such as afforestation and BECCS. Deploying CDR strategies with complementary operational characteristics highlights ERW as a key component of diversified climate mitigation portfolios.

While recent studies have begun to quantify the carbon transport potential of rivers in response to enhanced weathering inputs, such as Zhang et al. (2022), who modelled global thresholds for carbonate precipitation, and Harrington et al. (2023), who applied catchment‐specific simulations to UK basins, these efforts remain largely decoupled from detailed, mechanistic representations of terrestrial dynamics influencing ERW‐related processes. Expanding the framework to include catchment‐scale river processes would capture the downstream fate of weathering products from terrestrial feedstock dissolution to long‐term oceanic sequestration (Calabrese et al. 2022), enabling a more comprehensive assessment of ERW as a scalable CDR strategy. Ultimately, advancing integrated, process‐based modelling frameworks, such as T&C‐SMEW, is essential for quantifying whole‐ecosystem interactions and feedbacks, increasing confidence in CDR estimates, and guiding the effective deployment of ERW within broader climate mitigation efforts. These advances are key to positioning ERW as a robust and operationally feasible approach to achieving global CDR targets. As climate action timelines shorten, frameworks such as T&C‐SMEW will identify where ERW can deliver the greatest climate benefit.

## Author Contributions

**Ziyan Zhang:** conceptualization, investigation, methodology, validation, writing – review and editing, formal analysis. **Gregory Jones:** conceptualization, investigation, methodology, writing – review and editing, formal analysis, writing – original draft. **Salvatore Calabrese:** conceptualization, writing – review and editing. **Matteo Bertagni:** conceptualization, writing – review and editing. **Simone Fatichi:** conceptualization, software development, writing – review and editing. **Bonnie Waring:** funding acquisition, writing – review and editing, conceptualization, supervision. **Athanasios Paschalis:** conceptulization, funding acquisition, writing – review and editing, project administration, supervision.

## Funding

This work was supported by the Natural Environment Research Council, NE/Y000471/1; Foundation for Food and Agriculture Research, 22‐000070; USDA National Institute of Food and Agriculture (Hatch project 7010390).

## Conflicts of Interest

The authors declare no conflicts of interest.

## Supporting information


**Data S1:** gcb70650‐sup‐0001‐Supinfo.pdf.

## Data Availability

The data that support the findings of this study are openly available in Zenodo at https://zenodo.org/records/17699460.
