# Supplementary material for: An Integrated Modelling Framework to Determine Terrestrial Carbon Dioxide Removal via Enhanced Rock Weathering
Source: Glob Chang Biol. 2025 Dec 17;31(12):e70650. doi: 10.1111/gcb.70650 (PMC12710202; doi:10.1111/gcb.70650)
Supplement: Supplementary file 1 — Data S1: gcb70650‐sup‐0001‐Supinfo.pdf. [file GCB-31-e70650-s001.pdf]

Supplementary information for: An integrated modelling framework to determine terrestrial carbon dioxide removal via enhanced rock weathering

Ziyan Zhang [a,*]

Gregory Jones [b,*]

Salvatore Calabrese [c]

Matteo Bertagni [d]

Simone Fatichi [e]

Bonnie Waring [b]

Athanasios Paschalis [f]

[a] Department of Civil and Environmental Engineering, Imperial College London, London, SW7 2AZ, UK

[b] Department of Life Sciences, Imperial College London, Silwood Park, Ascot, SL5 7PY, UK

[c] Department of Biological and Agricultural Engineering, Texas A&M University, College Station, TX, 6 77843, USA

[d] Department of Environment, Land and Infrastructure Engineering, Politecnico di Torino, Turin, 10129, 8 Italy

[e] Department of Civil and Environmental Engineering, National University of Singapore, 117576, Singapore

[f] Department of Civil and Environmental Engineering, University of Cyprus, Nicosia, 20537, Cyprus

* Ziyan Zhang and Gregory Jones should be considered joint first authors

Contents

1 Water balance 3
2 Plant Nutrient Budget 4
2.1 Plant nutrient export 4
2.2 Plant stoichiometric constraints and flexibility 4
2.3 Plant nutrient uptake 6
2.4 Magnesium, calcium and potassium balances 7
2.5 Background inputs 7
2.6 Nutrient leaching 8
2.7 Alkalinity definition 8
3 Model parameters and input variables 9
3.1 Mesocosm setup description: Kelland et al. (2020) 9
3.2 Field setup description: Kantola et al. (2023)/ Beerling et al. (2024) 9
4 Uncertainty simulation setup in field experiment 13
5 Model spin-up procedure 15
6 Model comparison 18

1 Water balance

Soil surface water influx, q_{ins} (mm h^{-1}), can be calculated as the sum of direct rainfall on non-vegetated areas, throughfall for two vegetation layers, water released from the snowpack, drainage of intercepted water, and dew deposition. In spatially distributed simulations, runon is also considered part of q_{ins} and is determined as the total surface runoff from neighbouring elements contributing to the flow of a cell of interest (Fatichi et al., 2012).

To estimate soil moisture contents ϑ_i, the Richards equation is reduced to a system of ordinary differential equations. The soil column is discretised into a series of layers i = 1,…, n_s, by characterising each layer with a specific depth from the soil surface to the upper boundary layer, $Z_{s,i}$ (mm), layer thickness, $d_{z,i}$ (mm) and a distance between the centres of the current and preceding layer, $D_{z,i}$ (mm).

$$d_{z,i}\frac{d\theta_i}{dt} = q_{i-1} - q_i - \sum_{j=1}^{n_c} T_{H_v,j}\, r_{H_v,i,j} - \sum_{j=1}^{n_c} T_{L_v,j}\, r_{L_v,i,j} - \sum_{j=1}^{n_c} E_{g,j} - E_{bare} + Q_{l,in,i} + Q_{l,out,i} \tag{S1}$$

Where: q_i (mm h^{-1}) is the vertical flow from layer i, while terms with j denote moisture sinks within vegetated areas, and n_c is the number of vegetation types in the given element. Sinks at the soil surface and within the root zone result from evapotranspiration and can be divided into evaporation from bare soil, E_{bare} (mm h^{-1}), evaporation from the soil under the canopy, E_g (mm h^{-1}), and transpiration from high- and low-vegetation layers, T_{Hv}, and T_{Lv} (mm h^{-1}). Both E_g and E_{bare} only utilise moisture from the first soil layer. The influence of root biomass is displayed as r_i, representing the fraction of fine roots in each soil layer. Lateral outflows $Q_{l,out,i}$ (mm h^{-1}) are linked to the soil moisture content and estimations of the lateral head gradient. For a more detailed description of how the water budget is determined, such as estimations of infiltration excess runoff, saturation excess runoff, and subsurface flow (e.g., incoming subsurface lateral fluxes), refer to Fatichi et al. (2012).

2 Plant Nutrient Budget

The nutrient budget of the plant is obtained by computing changes in nutrient reserves of calcium, magnesium, and silicate $X_{reserve,TC}$ (g X m^{-2} VEG). Note that $m^{-2}\ VEG$ refer to the unit of projected canopy area. The reserve budgets are expressed as:

$$\frac{dX_{reserve,TC}}{dt} = X_{uptake,TC} - \frac{dTS_{X,TC}}{dt} - X_{export,l,TC} \tag{S2}$$

Where $X_{export,l,TC}$ (g X m^{-2} VEG day^{-1}) are the nutrients exported from living tissue through tissue turnover and X_{uptake} (g X m^{-2} VEG day^{-1}) are the fluxes of nutrients taken up from the soil. The changes in total nutrient content in the plant (e.g., $\frac{dTS_X}{dt}$) are a consequence of changes in the carbon content of the various pools. The total nutrient content of Ca, Mg, and Si $TS_{X,TC}$ (g X m^{-2} VEG) are given by:

$$TS_{X,TC} = \frac{C_{leaf}}{CX_l} + \frac{C_{sapw}}{CX_s} + \frac{C_{root}}{CX_r} + \frac{C_{hydr}}{CX_c} + \frac{C_{flfr}}{CX_f} + \frac{C_{heaw}}{CX_h} \tag{S3}$$

Where the carbon pools (g C m^{-2} VEG) are: green aboveground biomass (leaves) C_{leaf}, living sapwood (woody plants only) C_{sapw}, fine roots C_{root}, carbohydrate reserves C_{hydr}flower and fruits C_{flfr}, and heartwood (woody plants only) C_{heaw}. The terms CX_l, CX_s, CX_r, CX_c, CX_f, CX_h (g C g^{-1} X) are the target C: Ca/ Mg/ Si mass ratios of leaves, living sapwood, fine roots, carbohydrate reserves, flowers and fruits, and wood, respectively.

2.1 Plant nutrient export

The total flux of nutrients exported from living plant tissues $X_{export,l,TC}$ (g X m^{-2} VEG day^{-1}) are:

$$X_{export,l,TC} = r_{X_c}\left(1 - f_{transf,l}\right)\frac{S_{leaf}}{CX_l} + r_{X_c}\frac{S_{flfr}}{CX_f} + r_{X_c}\left(1 - f_{transf,r}\right)\frac{S_{root}}{CX_r} + \frac{S_{wood}}{CX_h} \tag{S4}$$

where S_{leaf}, S_{flfr}, S_{root}, S_{wood} (g C m^{-2} VEG) are the turnover rates of green aboveground biomass, fine roots, fruit and flowers, and wood, respectively, as computed by T&C (Fatichi et al., 2012).

2.2 Plant stoichiometric constraints and flexibility

Each plant tissue (C pool in the model abstraction) has a corresponding quantity of nutrients necessary for its growth and development. The target stoichiometric ratios

are prescribed quantities in the model and define the nutrients required for a plant with a balanced nutrient status for a given amount of carbon.

The maximum plant storage of Ca, Mg and Si $X_{sto,TC}$ (g X m^{-2} VEG) are computed considering a hypothetical stoichiometric flexibility of the carbon reserve pool, which defines a storage size:

$$X_{sto,TC} = \emptyset_s C_{hydr}\left(\frac{1}{CX_l} - \frac{1}{CX_c}\right) \tag{S5}$$

Where $\emptyset_s$ is a scaling parameter (Fatichi et al., 2012), assumed to be 1.

At each time step, the availability of Ca, Mg, and Si $X_{ava,TC}$ (g X m^{-2} VEG) to construct new tissue is given by:

$$X_{ava,TC} = X_{reserve,TC} + 0.35TNS_X \tag{S6}$$

Equation (S6) imposes a stoichiometric constraint on tissue construction, i.e., it can limit the allocation of NPP whenever the relative nutrient concentration of non-structural tissues r_{X_c} is dropping at the 0.65 level. In other words, when nutrient reserves in the model, $X_{reserve}$, are negative and equal to -0.35 of TNS_X, plant growth is limited by the availability of a specific nutrient. Note that the negative sign in $X_{reserve}$ is a modelling convenience and means a depletion of nutrients in non-structural tissues (e.g., r_{X_c}< 1).

The nutrients required, $X_{req,TC}$ (g X m^{-2} VEG) for the allocation of the unconstrained NPP is given by the allocation fraction to the different tissues minus the nutrient retained during tissue turnover:

$$\begin{aligned} X_{req,TC} = {} & f_l\frac{NPP}{CX_l} + f_f\frac{NPP}{CX_f} + f_c\frac{NPP}{CX_c} + f_r\frac{NPP}{CX_r} + f_s\frac{NPP}{CX_s} + \frac{T_{r_l}}{CX_l} \\ & + \frac{T_{r_r}}{CX_r} + \frac{S_{sapw}}{CX_h} - f_{transf,l}\frac{S_{leaf}}{CX_l} - f_{transf,r}\frac{S_{root}}{CX_r} \\ & - \frac{T_{r_C}}{CX_c} - \frac{S_{sapw}}{CX_s} - \frac{R_{exmy}}{CX_c} + \frac{Add_{AR}}{CX_c} \end{aligned} \tag{S7}$$

where f_i [−] are the allocation fractions for NPP (with i = l, f, c, r, s for leaves, flowers and fruits, carbohydrate reserves, fine roots, and living sapwood, respectively (Fatichi et al., 2012). S_{sapw} (g C m^{-2} VEG day^{-1}) is the amount of sapwood carbon biomass converted to heartwood, T_{r_l} and T_{r_r} (g C m^{-2} VEG day^{-1}) are the carbon translocation from reserves to leaves and fine roots, $T_{r_C} = T_{r_l} + T_{r_r}$, and $f_{transf,l}$ and $f_{transf,r}$ [−] are the fractions of nutrient resorption for leaves and fine roots. R_{exmy} (g C m^{-2} VEG day-

[1]) is the carbon exuded from roots and exported to mycorrhizal fungi and Add_{AR} (g C m^{-2} VEG day^{-1}) is the additional allocation to carbon reserve due to environmental constraints on growth (See T&C technical reference; Fatichi et al., 2012).

If X_{ava} are larger than X_{req} (the typical case), there are enough nutrients to allocate entirely NPP to the different tissues; otherwise, a reduction factor $f_{red,TC}$ [−] is computed for each nutrient as:

$$f_{red,TC} = \frac{\frac{X_{ava,TC}}{dt}}{X_{req,TC}} \tag{S8}$$

where *dt* is the time step in days, and NPP is corrected as NPP = f_{red}NPP. The remaining fraction of unallocated NPP is lost through autotrophic respiration as idling (or overflow) respiration, Ri = 1 – f_{red}NPP, because there is insufficient nutrient availability to build tissues. This case should be regarded as rare in natural conditions, but can occur in soils with poor nutrients or unfertilized crops where large quantities of nutrients are removed by harvesting fruits and seeds.

2.3 Plant nutrient uptake

Passive and active Base cation and Si uptake rates ($X_{uptake,TC}$) are determined per unit vegetation area (g *X* m^{-2} VEG day^{-1}) as:

$$X_{uptake,TC} = \frac{X_{up,TC}\left(1 - Sup_{x,TC}\right)}{Ccrown} \tag{S9}$$

$$X_{up,TC} = \max\left[X_{up,a,TC}, X_{up,p,TC}\right] \tag{S10}$$

$$Sup_{x,TC} = \frac{X_{reserve,TC} - 0.8X_{sto}}{L_{target}TNS_x + 0.2X_{sto}} \tag{S11}$$

Where: $X_{up,TC}$ represents the maximum of active ($X_{up,a,TC}$, g *X* m^{-2} day^{-1}) and passive uptake ($X_{up,p,TC}$, g *X* m^{-2} day^{-1}) of the base cation of interest (Mg, Ca, K) or Si with a soil solution concentration C_x (g *X* m^{-3}). C_{crown} is the area occupied by vegetation (m^2). $Sup_{x,TC}$ is a nutrient uptake suppression function that takes values between 1 (complete suppression) and 0 (no suppression), enabling plant nutrient uptake to be regulated depending on plant nutrient status. Nutrient uptake suppression begins when $X_{reserve,TC}$ is 80% of the maximum nutrient storage capacity for a respective nutrient, which prevents excessive nutrient uptake (e.g. when nutrients are (100*L_{target}) % above target nutrient stoichiometries (TNS_x)). The fraction L_{target} where full suppression of uptake occurs depends on the vegetation species.

Passive plant nutrient uptake is calculated as:

$$X_{up,p,TC} = a_x X_{tot,SMEW} \frac{T}{VT} \tag{S12}$$

Where a_x is the solubility coefficient for the respective nutrients, X (g X m^{-2}) is the available nutrient of interest, T (mm day^{-1}) is the transpiration flux from the biogeochemically active depth, VT (m) is the total water volume within the biogeochemically active layer. For Na, An and DIC, $a_X = 1$. For Al, $a_X = 0.001$; Ca, $a_X = 0.4$; Mg, $a_X = 0.4$; Si, $a_X = 0.001$.

$$X_{up,a,TC} = X_{up,r} + EM\, X_{up,em} + (1 - EM) X_{up.am} \tag{S13}$$

Where: $X_{up,r}$ represents root nutrient uptake, $X_{up,am}$ arbuscular mycorrhizal nutrient uptake and $X_{up,em}$ ectomycorrhizal nutrient uptake. EM is the ground area covered by plants associated with ectomycorrhiza. For a more detailed description of root and mycorrhizal nutrient uptake, refer to the supplementary material of Fatichi et al. (2019).

2.4 Magnesium, calcium and potassium balances

$$X_{tot,SMEW} = (n \cdot s \cdot Zbio[X] + \frac{f_X CEC}{z_X}) \cdot M_X \cdot n \cdot s \cdot Zbio \tag{S14}$$

$$[X] = \frac{X_{min,TC}}{M_X \cdot n \cdot s \cdot Zbio} \tag{S15}$$

Where: $X_{tot,SMEW}$ (g X m^{-2}) is the total amount of a specific cation within the biogeochemically active soil layer, n is the soil porosity, s denotes the soil moisture, $Zbio$ (m) is the biogeochemically active soil depth, and X is the concentration of the respective cation (mol m^{-3}). $f_X CEC$ represents the fraction of the total cation exchange capacity (CEC; mol m^{-2}) occupied by the cation of interest. z_X is the charge of the cation. The term $[X]$ represents the amount of cation X in the exchangeable pool (mol X m^{-3}). M_X (g mol^{-1}) is the molar mass of the cation X. The model assumes that a steady-state equilibrium is reached at each time step, ensuring that the concentration of base cations in solution remains constant during each time step.

2.5 Background inputs

To conserve element mass within the system, background inputs are determined using mean daily rates of leachate and plant transpiration scaled annually. These rates can be estimated from long-term field observations (e.g., Kantola et al., 2023).

$$I_{(Al,Na),TC} = \overline{\frac{L_k}{V} + \frac{T}{VT}} \cdot (Al, Na)_{tot,SMEW} \quad \text{(S16)}$$

Where: $I_{(Al,Na),TC}$ is a separate input term for Al and Na (g X m^{-2} day^{-1}). T (m day^{-1}) is the transpiration flux from the biogeochemistry active depth.

2.6 Nutrient leaching

The leaching approximation described in Fatichi et al. (2012) is assumed to hold, as most dissolved nutrients are typically located in the upper part of the soil column within the biogeochemically active zone. However, this approximation will primarily impact short-term nutrient leaching dynamics rather than long-term integrated leaching, where equilibrium is expected between leaching from the biogeochemically active zone and at the soil bottom. The equation for leaching ($L_{k,X,TC}$, m day^{-1}) is:

$$L_{k,X,TC} = a_X X_{tot} \cdot \frac{L_k}{V} \quad \text{(S17)}$$

Where X (g X m^{-2}) is the ion of interest. Refer above for additional equation notation details. a_X (−) are the solubility coefficients for the different solutes (Fatichi et al., 2019), Na, An and DIC, where $a_X = 1$. For Al, $a_X = 0.001$; Ca, $a_X = 0.4$; Mg, $a_X = 0.4$; Si, $a_X = 0.001$.

2.7 Alkalinity definition

T&C-SMEW adopts an explicit conservative definition of alkalinity based on Wolf-Gladrow (2007). We refer to conservative ions as those whose concentrations remain unchanged due to acid-base reactions, as described by Wolf-Gladrow et al. (2007). NH_4^+ is a non-conservative cation, as its concentrations can vary significantly over short timescales depending on biogeochemical processes and external inputs (Middelburg et al., 2020).

3 Model parameters and input variables

3.1 Mesocosm setup description: Kelland et al. (2020)

Irrigation occurred every 5 days, using a drip-feed system fixed at 12.5 mL min^{-1} for the duration of the experiment. The laboratory was controlled at 60-70% relative humidity and 25/ 17 °C day/ night temperatures. The daytime length was 18 hours for the first 60 days and 10 hours for the final 61 days. Photosynthetically active radiation was supplied to the plants and maintained at 800 μmol photons m^{-2} s^{-1}.

The meteorological forcings were set for both control and basalt addition simulations: hourly precipitation was set to match the reported irrigation amount over the experiment period, providing each irrigation event occurs within an hour, while the hourly air temperature was set to be the same as room-controlled temperature as reported. The air temperature was then used to derive dew temperature and vapour pressure deficit, given a constant relative humidity of 65%. Photosynthetically active radiation was set to 800 μmol photons m^{-2} s^{-1}, the wind speed was maintained at a constant value of 0.1 m s^{-1}, and the atmospheric CO_2 concentration was kept at 412 ppm. The initial soil pH was 6.6, and the cation exchange capacity (CEC) was fixed to be 25 cmol(+) kg $soil^{-1}$. The soil had a clay-loam texture, consisting of 31.8% clay and 32.8% sand. Soil organic matter (SOM) was estimated at 2.1%, based on soil organic C (SOC) by assuming a C content of 58%, following the van Bemmelen factor (1890). N fertiliser (180 kg N ha^{-1} in total) was dissolved in the irrigation water in the experiment and set at the constant rate of 0.82 g N m^{-2} for each irrigation (rainfall) event in the model. The initial base saturation and any other parameters followed the estimations used by Bertagni et al. (2025). The feedstock (Oregon basalt) mineralogy was represented as 35% labradorite, 23% alkali feldspar, 10% diopside, and 1% iron-rich forsterite (Lewis et al., 2021). The 3% apatite, identified by Lewis et al., (2021), was excluded due to its rapid dissolution in this mesocosm experiment. The remaining parameters required remained as default values (Fatichi et al., 2012a, Fatichi et al., 2012b; Fatichi et al., 2019; Kelland et al., 2020; Thermoddem, 2025).

3.2 Field setup description: Kantola et al. (2023)/ Beerling et al. (2024)

N fertiliser, 28% urea ammonium nitrate, was applied annually at 168 kg N ha^{-1} (assumed to be applied at a constant rate of 0.84 g N m^{-2} for 20 days) before maize planting. Forcings and climate inputs were obtained from ERA5-Land (Muñoz Sabater, 2019). The soil texture was a silty loam consisting of 35% clay, 10% sand, and 2.5% SOM based on SoilGrids (ISRIC, 2020). Due to the depth-averaged framework of the soil biogeochemistry module, weathering and nutrient dynamics are lumped within the active layer of 30 cm and assumed to be vertically homogeneous.

The model was first setup for the pretreatment period (2009-2016), to represent the ecosystem C fluxes. The sowing and harvest dates were informed by NEE data measured from local flux tower. Due to the lack of openly available data for the duration of the experiment, plot digitizer software (Ankit, 2024) was used to extract flux tower observations from Kantola et al. (2023). The initial values in soil biogeochemistry module, such carbon and nutrient pools, and initial tissue nutrient reserves, were estimated from model spin-up procedure (Supplementary Information Section 5). The remaining parameters required remained as default values (Fatichi et al., 2012a, Fatichi et al., 2012b; Fatichi et al., 2019; Lewis et al., 2021; Thermoddem, 2025).

Table S1: Major forcings, input variables and parameters used to initialise model simulations, separated into meteorological inputs, plant functional type/ rotation, soil physicochemical and biogeochemical parameters, fertiliser application and feedstock characterisation.

Relevant Model Inputs/Parameters		Units	Kelland et al., 2020	Kantola et al., 2023/ Beerling et al., 2024	Notes
meteorological inputs	relative humidity	—	0.65	ERA5-Land hourly dataset	
	air temperature	°C	25 (day)/ 17(night)		
	PAR (diffused)	W m^{-2}	173.6		
	precipitation	mm hr^{-1}	equivalent to irrigation (values in Figure S2)		
	atmospheric CO_2	ppm	412	400 - 420	
plant functional type/ crop rotation		—	C4	Maize–maize–soybean–maize	Prescribed parameters adapted from previous model applications for relevant species (Mastrotheodoros et al., 2017; Paschalis et al., 2018; Moustakis et al., 2022)
soil texture	clay, sand	%	31.8, 32.8	35, 10	From experimental measurements, or Soil Grids (ISRIC, 2020)
organic matter		%	2.1	2.5	
soil CEC		cmol(+) kg^{-1}	25.4	8.6	
dry soil density		kg m^{-3}	1125	1295	Model based on Kelland et al., (2020) and Kantola et al., (2023)
biogeochemically active soil layer depth		mm	500	300	Based on experiment measurement layer depth
initial pH		—	6.6	6.1	
CEC fractions (Ca, Mg, K, Na, Al, H)			[0.8871 0.0390 0.0561 0.0022 0.0000 0.0155]	[0.0027 0.012 0.022 0.76 0.05 0.15]	From experimental measurements, or model spin-up procedure

						(Supplementary Information Section 5)
Annual N fertiliser application		gN m^{-2}	18	5.6		
Basalt application rate		kg m^{-2}	10	5 every year in November		
Feedstock composition	mineralogy	—	["labradorite", "alkali feldspar", "diopside", "Fe forsterite"]	["albite", "ferroactinolite, "epidote", "chlorite","quartz"] (Lewis et al., 2021)	["albite", "ferroactinolite", "epidote", "chlorite", "quartz"," calcite"] (Beerling et al., 2024)	Kinetic parameters from experiment papers, or available datasets (Palandri & Kharaka, 2004; Libes, 2011; Thermoddem, 2025)
	mineralogy stoichiometry	—	[0.35, 0.23, 0.1, 0.01]	[0.196,0.116, 0.256,0.363, 0.052]	[0.233,0.119, 0.178,0.34, 0.09,0.026]	
Feedstock Specific surface area		m^2 g^{-1}	7.35	1.02		Keland et al., (2020) and Lewis et al., (2021)
Dissolution factor		—	0.5	1		

4 Uncertainty simulation setup in field experiment

To explore the uncertainty in model parameters and its influence on simulated carbon dioxide removal potential (CDR) in a real world, key parameters and variables in soil biogeochemistry, hydrology and weathering module were selected to vary their original values within a reasonable minimum/maximum range, as summarized in Table S2.

A version of T&C-BG (i.e., without enhanced rock weathering module) was firstly run with a set of simulations, in which 12 soil biogeochemistry parameters were altered individually and all together, yielding a plausible range of modelled soil respiration from 27 simulations in total (i.e., 1 simulation with original values+2 simulations*12 parameters altered individually+2 simulations when all parameters were altered at the same time). Therefore, from these simulations, the maximum and minimum time-series soil respiration were obtained.

The ERW module was then run independently from T&C-SMEW, with the uncertainty ranges in soil respiration, effective soil pore saturation and dissolution factor as stated in Table S2. All other dynamics incorporated from T&C-BG (e.g., vegetation uptakes) were assumed to be the same as the original simulations. Therefore, 27 simulations were obtained for $Basalt_L$ and $Basalt_B$ scenarios respectively, providing a plausible range in element concentrations, weathering rates as well as CDR estimates from the model as shown in Figure 7.

Table S2: List of selected parameters and variables in uncertainty analysis.

Parameters/ Variables	Description	Original	Min.	Max.	Associated Dynamics
**Vm**	maximum specific decomposition rate for MOC	100	1.2	528	Soil respiration
**Vpc**	maximum specific decomposition rate of POC - cellulose/hemicellulose-pectin	200	4.8	792	
**Km**	half-saturation constant for decomposition of MOC	0.25	0.057	0.95	
**rem**	turnover rate of ectomycorrhizal fungi	0.018	0.0075	0.075	
**rep**	turnover rate of extracellular enzyme	0.018	0.0075	0.075	
**pepf**	fraction of maintenance respiration for production of enzymes for POC from fungi	0.006	0.0031	0.031	
**mrb**	specific maintenance factor or rate of bacteria	0.005	0.0012	0.03	
**mrf**	specific maintenance factor or rate of saprotrophic fungi	0.002	0.0012	0.03	
**fd**	fraction of decomposed POC allocated to DOC	0.4	0.2	0.8	
**Vpl**	maximum specific decomposition rate of POC-lignin	23	4.8	792	
**Vdb**	maximum specific uptake rate of DOC for growth of bacteria	0.04	0.0024	0.06	
**Vdf**	maximum specific uptake rate of DOC for growth of saprotrophic fungi	0.02	0.0024	0.06	
**Rhet +Raut**	Soil respiration (heterotrophic + autotrophic)	Simulated from T&C-BG via varying above parameters			Soil inorganic carbon pool

**s**	Effective soil pore saturation	Simulated from T&C-SMEW	s^2	sqrt(s)	Weathering rate; Hydrology
**F_D**	Dissolution factor	1	0.8	1.2	Weathering rate

5 Model spin-up procedure

The model spin-up procedure is adapted from the methodology described in Luo et al. (2024), targeting 78 pools in the soil biogeochemistry module (expanding on the 55 pools of Fatichi et al., 2019). This is a common practice to set up initial conditions in soil biogeochemistry models and stabilise the biogeochemical fluxes (e.g., Liao et al., 2023).

The spin-up procedure was implemented using a control setup (i.e., no basalt application). The identified steady-state soil carbon and nutrient pool values from this procedure were used as the same initial condition of soil biogeochemistry pool for both control and basalt simulations in the model.

The full procedure follows three main steps. The T&C-SMEW model was run first without the ERW and soil biogeochemistry modules to obtain nutrient-unstressed water and vegetation fluxes with the site-specific setup. The site-specific setup includes local meteorological forcings, vegetation cover, vegetation phenology parameters and crop related parameters (Fatichi et al., 2019; Buckley Paules et al., 2025). The crop rotation setup follows the work of Buckley Paules et al. (2025), which was applied in Nebraska, USA (AmeriFlux site US-NE1).

The long-term averages (constants) of these outputs were used to run the soil biogeochemistry module independently. This phase continued until all soil carbon and nutrient pools reached a steady state (i.e., values no longer changed over time after a transient period); the specific duration required was not predefined but depended entirely on achieving system stability (typically within 100-1000 simulation years).

The model then was run without ERW module for the simulation period (i.e., year 2009-2020) as a loop until reaching the steady state of a dynamic equilibrium in soil biogeochemistry pools. Figure S1 shows an example of a steady-state soil organic carbon pool as well as heterotrophic respiration in the 12-year spin-up simulation. Once all carbon and nutrient pools reach a steady state, the average values of each pool in the last-year simulation result were used as initial conditions in soil biogeochemistry module for further simulations. While using those initial conditions, the fully-coupled version of T&C-SMEW model was also tested to provide a final check on the initial ion charge balance.

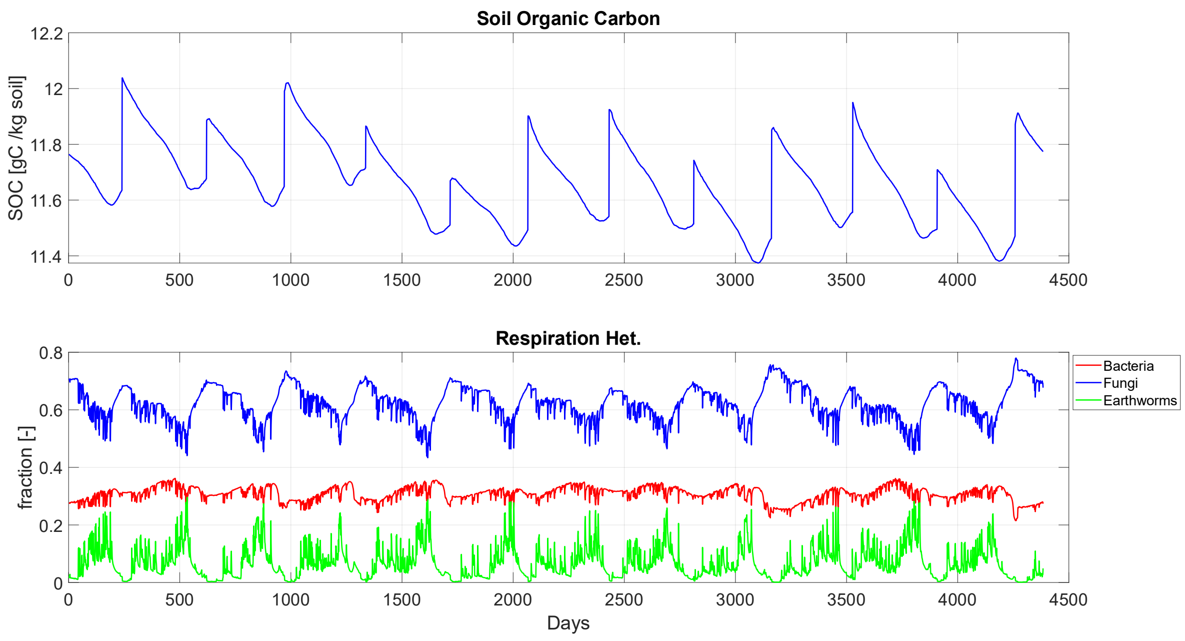

Figure S1: An example of a steady-state soil organic carbon pool as well as heterotrophic respiration in the 10-year spin-up simulation during pretreatment years.

Table S3: The 78 carbon and nutrient pools simulated by the soil biogeochemistry module.

Pool	Description
Carbon	Above-ground Litter Metabolic
	Above-ground Litter Structural - Cellulose/Hemicellulose
	Above-ground Litter Structural - Lignin
	Above-ground Woody - Cellulose/Hemicellulose
	Above-ground Woody - Lignin
	Below-ground Litter Metabolic
	Below-ground Litter Structural - Cellulose/Hemicellulose
	Below-ground Litter Structural - Lignin
	SOM-POC - Lignin
	SOM-POC - Cellulose/Hemicellulose
	SOM-MOC
	DOC - for bacteria
	DOC - for fungi
	Enzyme for decomposition of POC - Bacteria
	Enzyme for decomposition of POC - Fungi
	Enzyme for decomposition of MOC - Bacteria
	Enzyme for decomposition of MOC - Fungi
	Bacteria pool
	Fungi saprotrophic
	AM-Mycorrhizal - C
	EM-Mycorrhizal - C
	Earthworms - C
Nitrogen	Nitrogen Above-ground Litter
	Nitrogen Above-ground Woody
	Nitrogen Below-ground Litter
	Nitrogen SOM
	Nitrogen Bacteria
	Nitrogen Fungi
	AM-Mycorrhizal - N
	EM-Mycorrhizal - N
	Nitrogen Ion Ammonium NH_4^+
	Nitrogen Nitrate NO_3^-
	DON

	Earthworms - N
Phosphorus	Phosphorus Above-ground Litter<br>Phosphorus Above-ground Woody<br>Phosphorus Below-ground Litter<br>Phosphorus SOM<br>Phosphorus Bacteria<br>Phosphorus Fungi<br>AM-Mycorrhizal - P<br>EM-Mycorrhizal - P<br>Phosphorus Mineral<br>Phosphorus Primary<br>Phosphorus Secondary<br>Phosphorus Occluded<br>DOP
Potassium	Potassium Above-ground Litter<br>Potassium Above-ground Woody<br>Potassium Below-ground Litter<br>Potassium SOM<br>Potassium Mineral Solution<br>Potassium Exchangeable<br>Potassium Fixed or Non-exchangeable<br>Potassium in Lattice of Primary Minerals
Calcium	Calcium Above-ground Litter<br>Calcium Above-ground Woody<br>Calcium Below-ground Litter<br>Calcium SOM<br>Calcium Mineral Solution<br>Calcium Exchangeable<br>Calcium Fixed or Non-exchangeable<br>Calcium in Lattice of Primary Minerals
Magnesium	Magnesium Above-ground Litter<br>Magnesium Above-ground Woody<br>Magnesium Below-ground Litter<br>Magnesium SOM<br>Magnesium Mineral Solution<br>Magnesium Exchangeable<br>Magnesium Fixed or Non-exchangeable<br>Magnesium in Lattice of Primary Minerals
Silicate	Silicate Above-ground Litter<br>Silicate Above-ground Woody<br>Silicate Below-ground Litter<br>Silicate SOM<br>Silicate Mineral Solution<br>Silicate Fixed or Non-exchangeable<br>Silicate in Lattice of Primary Minerals

6 Model comparison

Table S4: Summary of feedstock element release (mmol m^{-2} d^{-1}), pH, and CO_2 sequestration (g m^{-2} d^{-1}) from Kelland et al. (2020): observations and model estimates with corresponding differences between observed and modelled mean values, standard errors (SE), reported to two significant figures.

**Component**		**Observed mean**	**Observed SE**	**ΔT&C-SMEW Model mean**	**ΔSMEW Model mean**
Feedstock element release (mmol m^{-2} d^{-1}) [Kelland et al. (2020) Table 2]	Ca	18	14	-2	0.0
	Mg	4.2	0.90	-0.9	+1.8
	K	1.1	1.4	-1.0	-1.1
	Na	0.3	0.50	+4.5	+5.4
	Si	1.9	0.40	+34	+42
pH [Kelland et al. (2020) Figure 3e]	No basalt	6.6	0.050	-0.07	-0.2
	Basalt	6.7	0.050	+0.08	+0.2
CO_2 sequestration (g m^{-2} d^{-1}) [Kelland et al. (2020) Table 2]	Potential	2.0	1.4	-0.09	+0.4
	Effective	-0.0010	0.010	+0.06	+0.4

Table S5: Summary of the soil nutrient budget (g m^{-2}), partitioned into leachate, bulk soil and plant components from Kelland et al. (2020): observations and model estimates with corresponding differences between observed and modelled mean values, reported to two significant figures.

Component				**Observed mean**	**Observed SE**	**ΔT&C-SMEW model mean**	**ΔSMEW Model mean**
Nutrient budget (g m^{-2})	Cumulative leachate [Kelland et al. (2020) Figure 3]	No basalt	Ca	7.5	0.28	-1.9	+6.5
			Mg	0.075	0.0050	+0.27	+0.63
			Si	0.75	0.015	-0.75	-0.56
		Basalt	Ca	7.5	0.21	+0.25	+20
			Mg	0.075	0.0025	+0.46	+1.7
			Si	0.75	0.020	-0.66	+67
	Soil column [Kelland et al. (2020) Figure 3]	No basalt	Ca	2700	0.010	-220	-500
			Mg	79	1.9	-16	-33
			Si	4.7	0.25	-4.7	+4.5
		Basalt	Ca	2800	65	-250	-600
			Mg	89	1.9	-17	-28
			Si	5.8	0.20	+92	+30
	Plant [Kelland et al. (2020) Figure 2]	No basalt	Ca	13	0.25	-1.5	-3.8
			Mg	4.0	0.20	-0.4	-3.5
			Si	20	0.94	-10	-19
		Basalt	Ca	15	0.0050	-3	+3.0
			Mg	4.5	0.11	-0.87	-3.3
			Si	25	0.85	-4	+21
	Total	No basalt	Ca	2700	0.010	-220	-500
			Mg	83	1.9	-16	-19
			Si	25	1.0	-16	+125
		Basalt	Ca	2800	65	-250	-600
			Mg	94	1.9	-18	-47
			Si	31	0.85	+87	-30

Table S6: Summary of the ecosystem C flux (g m^{-2}): observations and model estimates with corresponding mean values, reported to two significant figures. The model results correspond to the default set up without uncertainty simulations.

Component				Observed mean	Model mean		
					No Basalt	L	B
Ecosystem flux (g m^{-2} d^{-1}) [Kantola et al. (2023) Figure 1]	Maize	No basalt	GPP	17	14	-	-
			NEE	-6.8	-4.2	-	-
			R_e	9.6	10	-	-
		Basalt	GPP	16	-	14	14
			NEE	-6.4	-	-4.2	-4.2
			R_e	9.3	-	10	10
	Soybean	No basalt	GPP	11	9.4	-	-
			NEE	-3.6	-3.5	-	-
			R_e	7.1	5.8	-	-
		Basalt	GPP	8.7	-	9.4	9.4
			NEE	-3.5	-	-3.5	-3.5
			R_e	5.3	-	5.8	5.8

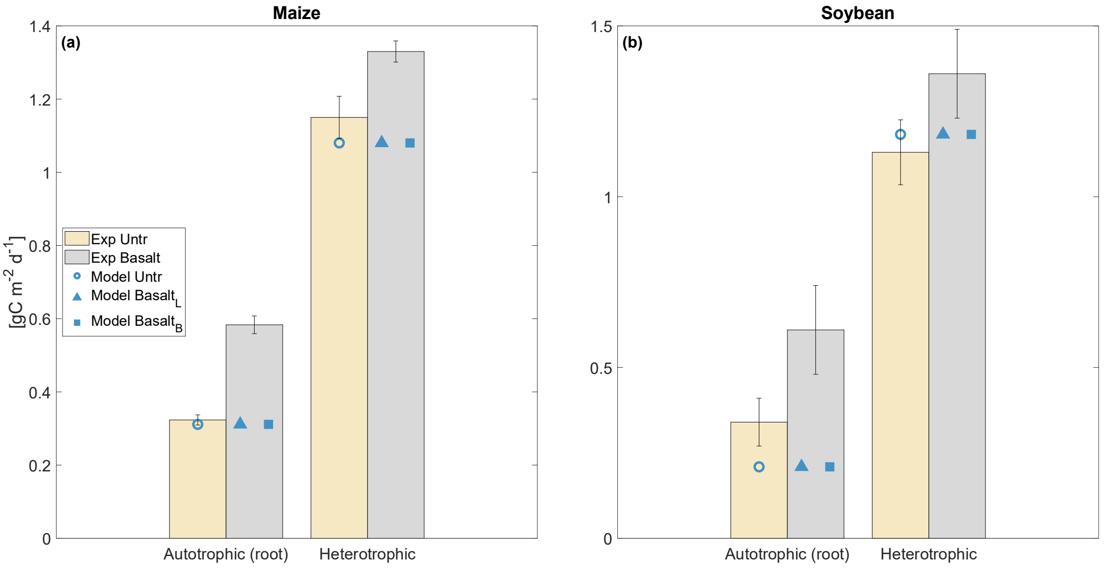

Figure S2: (a) Maize (2017, 2018, 2020) and (b) soybean (2019) autotrophic (root) and heterotrophic respiration (gC m^{-2} d^{-1}) averaged across experimental years. Grey bars represent mean experimental values from basalt treatments, and orange bars from control/ no basalt treatments (Kantola et al., 2023). Error bars represent standard errors from experiment measurements among all plots. Triangular points correspond to simulated values using the Lewis et al. (2021) feedstock characterisation and square points to the Beerling et al. (2024) characterisation. The subscripts L and B relate to feedstock mineralogy from Lewis et al. (2021) and Beerling et al. (2024), respectively.

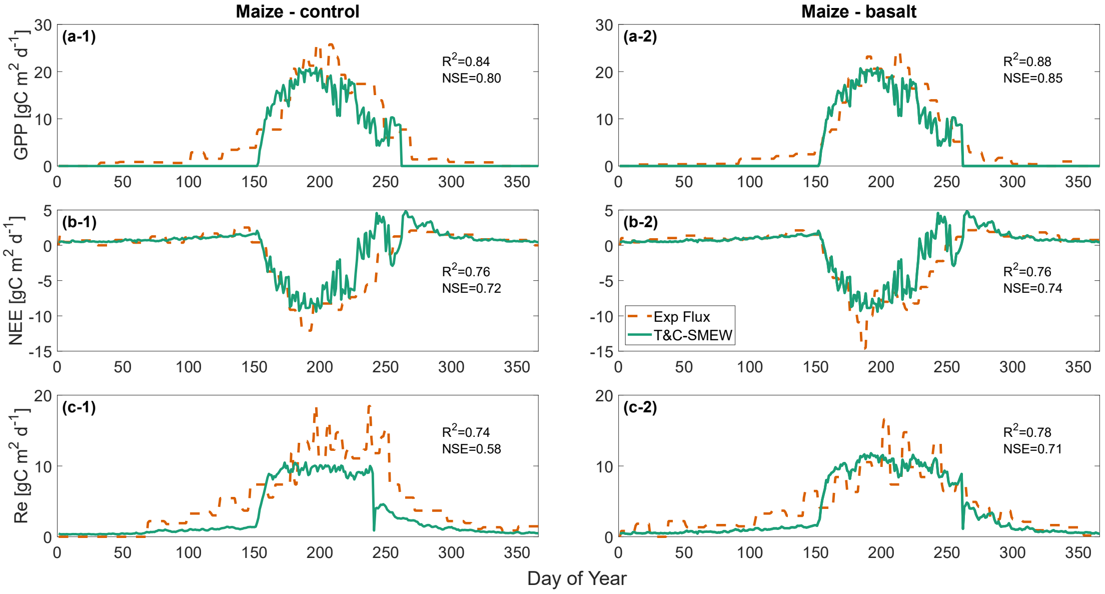

Figure S3: Annual time series of average daily ecosystem carbon fluxes for the maize rotation years (2017, 2018, and 2020) (Kantola et al., 2023). Carbon fluxes are partitioned into (a) gross primary production (GPP), (b) net ecosystem exchange (NEE), and (c) ecosystem respiration (Re) (g m^{-2} d^{-1}), and are shown separately for control (a, b, c-1) and basalt-amended (a, b, c-2) treatments. Solid green lines represent simulated values, while dotted lines denote observations extracted from the AmeriFlux network. Simulated NEE values were tuned to match the seasonality of observations.

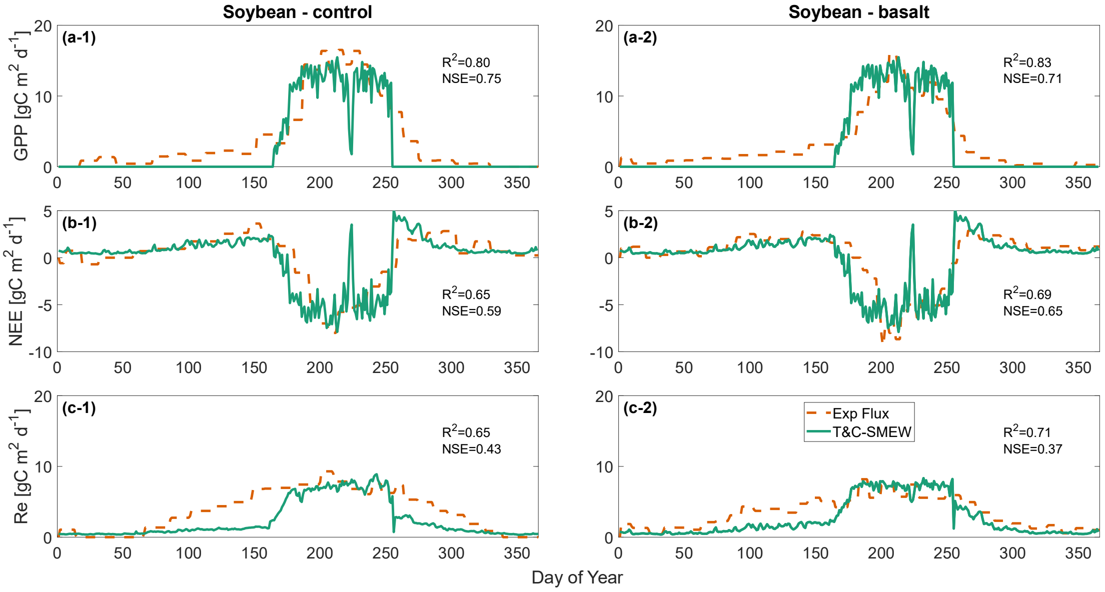

Figure S4: Annual time series of average daily ecosystem carbon fluxes for the soybean rotation year (2019) (Kantola et al., 2023). Carbon fluxes are partitioned into (a) gross primary production (GPP), (b) net ecosystem exchange (NEE), and (c) ecosystem respiration (Re) (g m^{-2} d^{-1}), and are shown separately for control (a, b, c-1) and basalt-amended (a, b, c-2) treatments. Solid green lines represent simulated values, while dotted lines denote observations extracted from the AmeriFlux network. Simulated NEE values were tuned to match the seasonality of observations.

Table S7: Summary of the soil pH and base saturation (%): observations (Beerling et al., 2024) and model estimates with corresponding mean values and standard errors (SE), reported to two significant figures. L mineralogy corresponds to the feedstock characterised by Lewis et al. (2021), while B mineralogy is that from Beerling et al. (2024). The model results correspond to the default set up without uncertainty simulations.

Component			Observed mean (0-10 cm)	Observed se (0-10 cm)	Observed mean (10-30 cm)	Observed se (10-30 cm)	Model mean		
							No Basalt	L	B
Soil pH [Beerling et al. (2024) Appendix 01 Figure S6]	No basalt	Pre	6.2	0.06	6	0.11	6.1	-	-
		2017	6.3	0.074	6.3	0.081	6	-	-
		2018	6.3	0.063	6.3	0.077	6.1	-	-
		2019	6.1	0.077	6.1	0.074	6.1	-	-
		2020	6	0.055	6.2	0.074	6	-	-
	Basalt	Pre	6.1	0.077	6.1	0.07	-	6.1	6.1
		2017	6.6	0.055	6.4	0.059	-	6.1	6.3
		2018	6.7	0.04	6.6	0.052	-	6.3	6.6
		2019	6.9	0	6.5	0.05	-	6.6	6.8
		2020	6.9	0	6.5	0.05	-	7	7
Base saturation (%) [Beerling et al. (2024) Appendix 01 Figure S6]	No basalt	Pre	80	1	81	1.6	79	-	-
		2017	81	1	77	2.1	80	-	-
		2018	85	1.2	77	1.8	80	-	-
		2019	79	1	76	1	79	-	-
		2020	73	0	83	1.9	80	-	-
	Basalt	Pre	81	1	83	1.4	-	79	79
		2017	83	0	80	1	-	82	89
		2018	90	1.4	81	1	-	88	95
		2019	88	0	81	0	-	95	97
		2020	83	0	87	1.4	-	98	98

Table S8: Summary of feedstock element release (mmol m^{-2}) and cumulative potential CO_2 removal (CDR) (t CO_2 ha^{-1}): observations (Beerling et al., 2024) and model estimates with corresponding mean values and standard error of the mean (SE), reported to two significant figures. L mineralogy corresponds to the feedstock characterised by Lewis et al. (2021), while B mineralogy is that from Beerling et al. (2024). The model results correspond to the default set up without uncertainty simulations.

Component			Observed mean	Observed SE	Model (L) mean	Model (B) mean
Feedstock cation release (mmol m^{-2}) [Beerling et al. (2024) Dataset S06]	Ca	2017	2300	390	330	1600
		2018	6300	370	1100	3400
		2019	9900	350	2100	3800
		2020	6900	690	2900	3500
	Mg	2017	2100	240	560	560
		2018	1400	560	1900	1900
		2019	3200	720	3600	3500
		2020	5100	820	5100	5000
Cumulative potential CDR (t CO_2 ha^{-1}) [Beerling et al. (2024) Dataset S06]		2017	3.8	1.8	0.78	1.9
		2018	6.8	2.5	2.6	4.7
		2019	11	3.3	4.9	6.4
		2020	11	3.7	7.1	7.5

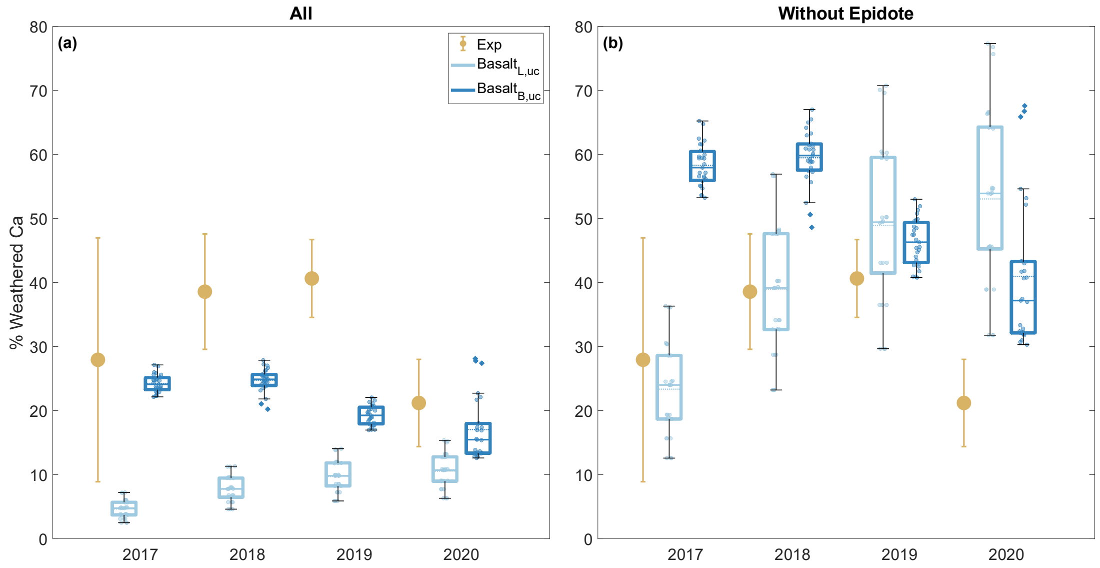

Figure S5: The percent of weathered Ca from the feedstock (a) when considering all minerals, and (b) without epidote in the simulations (blue), compared to the estimated weathered Ca percentage in experimental measurement (orange). Error bars for experiamental data represent ± one standard deviation (SD).

Table S9: Summary of plant nutrient contents (g m^{-2}) observations (Beerling et al., 2024) and model estimates with corresponding mean values and standard errors (SE), reported to two significant figures. L mineralogy corresponds to the feedstock characterised by Lewis et al. (2021), while B mineralogy is that from Beerling et al. (2024). The model results correspond to the default set up without uncertainty simulations.

**Component**				**Observed mean**	**Observed SE**	**Modelled Mean**		
						No Basalt	L	B
Plant nutrient contents (g m^{-2}) [Kantola et al. (2023) Appendix S2]	Maize	No basalt	Ca	3.7	1.1	3.7	-	-
			Mg	2.9	0.99	3	-	-
		Basalt	Ca	4.4	1.4	-	5.1	5.2
			Mg	3.3	1.1	-	3.1	3.1
	Soybean	No basalt	Ca	8.1	1.2	7.1	-	-
			Mg	3.2	0.65	3.3	-	-
		Basalt	Ca	8.5	1.5	-	10	10
			Mg	3.5	1.3	-	3.3	3.3
